# ENIGMA and global neuroscience: A decade of large-scale studies of the brain in health and disease across more than 40 countries

**DOI:** 10.1038/s41398-020-0705-1

**Published:** 2020-03-20

**Authors:** Paul M. Thompson, Neda Jahanshad, Christopher R. K. Ching, Lauren E. Salminen, Sophia I. Thomopoulos, Joanna Bright, Bernhard T. Baune, Sara Bertolín, Janita Bralten, Willem B. Bruin, Robin Bülow, Jian Chen, Yann Chye, Udo Dannlowski, Carolien G. F. de Kovel, Gary Donohoe, Lisa T. Eyler, Stephen V. Faraone, Pauline Favre, Courtney A. Filippi, Thomas Frodl, Daniel Garijo, Yolanda Gil, Hans J. Grabe, Katrina L. Grasby, Tomas Hajek, Laura K. M. Han, Sean N. Hatton, Kevin Hilbert, Tiffany C. Ho, Laurena Holleran, Georg Homuth, Norbert Hosten, Josselin Houenou, Iliyan Ivanov, Tianye Jia, Sinead Kelly, Marieke Klein, Jun Soo Kwon, Max A. Laansma, Jeanne Leerssen, Ulrike Lueken, Abraham Nunes, Joseph O’ Neill, Nils Opel, Fabrizio Piras, Federica Piras, Merel C. Postema, Elena Pozzi, Natalia Shatokhina, Carles Soriano-Mas, Gianfranco Spalletta, Daqiang Sun, Alexander Teumer, Amanda K. Tilot, Leonardo Tozzi, Celia van der Merwe, Eus J. W. Van Someren, Guido A. van Wingen, Henry Völzke, Esther Walton, Lei Wang, Anderson M. Winkler, Katharina Wittfeld, Margaret J. Wright, Je-Yeon Yun, Guohao Zhang, Yanli Zhang-James, Bhim M. Adhikari, Ingrid Agartz, Moji Aghajani, André Aleman, Robert R. Althoff, Andre Altmann, Ole A. Andreassen, David A. Baron, Brenda L. Bartnik-Olson, Janna Marie Bas-Hoogendam, Arielle R. Baskin-Sommers, Carrie E. Bearden, Laura A. Berner, Premika S. W. Boedhoe, Rachel M. Brouwer, Jan K. Buitelaar, Karen Caeyenberghs, Charlotte A. M. Cecil, Ronald A. Cohen, James H. Cole, Patricia J. Conrod, Stephane A. De Brito, Sonja M. C. de Zwarte, Emily L. Dennis, Sylvane Desrivieres, Danai Dima, Stefan Ehrlich, Carrie Esopenko, Graeme Fairchild, Simon E. Fisher, Jean-Paul Fouche, Clyde Francks, Sophia Frangou, Barbara Franke, Hugh P. Garavan, David C. Glahn, Nynke A. Groenewold, Tiril P. Gurholt, Boris A. Gutman, Tim Hahn, Ian H. Harding, Dennis Hernaus, Derrek P. Hibar, Frank G. Hillary, Martine Hoogman, Hilleke E. Hulshoff Pol, Maria Jalbrzikowski, George A. Karkashadze, Eduard T. Klapwijk, Rebecca C. Knickmeyer, Peter Kochunov, Inga K. Koerte, Xiang-Zhen Kong, Sook-Lei Liew, Alexander P. Lin, Mark W. Logue, Eileen Luders, Fabio Macciardi, Scott Mackey, Andrew R. Mayer, Carrie R. McDonald, Agnes B. McMahon, Sarah E. Medland, Gemma Modinos, Rajendra A. Morey, Sven C. Mueller, Pratik Mukherjee, Leyla Namazova-Baranova, Talia M. Nir, Alexander Olsen, Peristera Paschou, Daniel S. Pine, Fabrizio Pizzagalli, Miguel E. Rentería, Jonathan D. Rohrer, Philipp G. Sämann, Lianne Schmaal, Gunter Schumann, Mark S. Shiroishi, Sanjay M. Sisodiya, Dirk J. A. Smit, Ida E. Sønderby, Dan J. Stein, Jason L. Stein, Masoud Tahmasian, David F. Tate, Jessica A. Turner, Odile A. van den Heuvel, Nic J. A. van der Wee, Ysbrand D. van der Werf, Theo G. M. van Erp, Neeltje E. M. van Haren, Daan van Rooij, Laura S. van Velzen, Ilya M. Veer, Dick J. Veltman, Julio E. Villalon-Reina, Henrik Walter, Christopher D. Whelan, Elisabeth A. Wilde, Mojtaba Zarei, Vladimir Zelman, for the ENIGMA Consortium

**Affiliations:** 1 Imaging Genetics Center, Mark and Mary Stevens Neuroimaging and Informatics Institute, Keck School of Medicine, University of Southern California, Marina del Rey, CA USA; 2 Department of Psychiatry, University of Münster, Münster, Germany; 3 Department of Psychiatry, The University of Melbourne, Melbourne, VIC Australia; 4 The Florey Institute of Neuroscience and Mental Health, The University of Melbourne, Melbourne, VIC Australia; 5 Department of Psychiatry, Bellvitge University Hospital, Bellvitge Biomedical Research Institute-IDIBELL, Barcelona, Spain; 6 Department of Human Genetics, Radboud University Medical Center, Nijmegen, The Netherlands; 7 Donders Institute for Brain, Cognition and Behaviour, Radboud University, Nijmegen, The Netherlands; 8 Department of Psychiatry, Amsterdam UMC, University of Amsterdam, Amsterdam Neuroscience, Amsterdam, The Netherlands; 9 Institute for Diagnostic Radiology and Neuroradiology, University Medicine Greifswald, Greifswald, Germany; 10 Department of Computer Science and Engineering, The Ohio State University, Columbus, OH USA; 11 Turner Institute for Brain and Mental Health, School of Psychological Sciences, Monash University, Clayton, VIC Australia; 12 Biometris Wageningen University and Research, Wageningen, The Netherlands; 13 Language & Genetics Department, Max Planck Institute for Psycholinguistics, Nijmegen, The Netherlands; 14 The Center for Neuroimaging and Cognitive Genomics, School of Psychology, National University of Ireland, Galway, Ireland; 15 Department of Psychiatry, University of California, San Diego, La Jolla, CA USA; 16 Desert-Pacific Mental Illness Research, Education, and Clinical Center, VA San Diego Healthcare System, San Diego, CA USA; 17 Departments of Psychiatry and of Neuroscience and Physiology, SUNY Upstate Medical University, Syracuse, NY USA; 18 INSERM Unit 955 Team 15 ‘Translational Psychiatry’, Créteil, France; 19 NeuroSpin, UNIACT Lab, Psychiatry Team, CEA Saclay, Gif-Sur-Yvette, France; 20 National Institute of Mental Health, National of Health, Bethesda, MD USA; 21 Department of Psychiatry and Psychotherapy, Otto von Guericke University Magdeburg, Magdeburg, Germany; 22 Department of Psychiatry, Trinity College Dublin, Dublin, Ireland; 23 German Center for Neurodegenerative Diseases (DZNE), Magdeburg, Germany; 24 Information Sciences Institute, University of Southern California, Marina del Rey, CA USA; 25 Department of Computer Science, University of Southern California, Los Angeles, CA USA; 26 Department of Psychiatry and Psychotherapy, University Medicine Greifswald, Greifswald, Germany; 27 German Center for Neurodegenerative Diseases (DZNE), Site Rostock/Greifswald, Greifswald, Germany; 28 Psychiatric Genetics, QIMR Berghofer Medical Research Institute, Brisbane, QLD Australia; 29 Department of Psychiatry, Dalhousie University, Halifax, NS Canada; 30 National Institute of Mental Health, Klecany, Czech Republic; 31 Department of Psychiatry, Amsterdam University Medical Centers, VU University Medical Center, GGZ inGeest, Amsterdam Neuroscience, Amsterdam, The Netherlands; 32 Center for Multimodal Imaging and Genetics, University of California, San Diego, La Jolla, CA USA; 33 Brain and Mind Centre, University of Sydney, Sydney, Australia; 34 Department of Psychology, Humboldt-Universität zu Berlin, Berlin, Germany; 35 Department of Psychiatry & Behavioral Sciences, Stanford University, Stanford, CA USA; 36 Department of Psychiatry & Weill Institute for Neurosciences, University of California, San Francisco, San Francisco, CA USA; 37 Interfaculty Institute for Genetics and Functional Genomics, University Medicine Greifswald, Greifswald, Germany; 38 APHP, Mondor University Hospitals, School of Medicine, DMU Impact, Psychiatry Department, Créteil, France; 39 Icahn School of Medicine at Mount Sinai, New York, NY USA; 40 Institute of Science and Technology for Brain-Inspired Intelligence, Fudan University, Shanghai, China; 41 MOE Key Laboratory of Computational Neuroscience and Brain-Inspired Intelligence, Fudan University, Shanghai, China; 42 Centre for Population Neuroscience and Precision Medicine (PONS), MRC SGDP Centre, Institute of Psychiatry, Psychology & Neuroscience, King’s College London, London, UK; 43 Department of Psychiatry, Beth Israel Deaconess Medical Center, Harvard Medical School, Boston, MA USA; 44 Department of Psychiatry, Brigham and Women’s Hospital, Boston, MA USA; 45 Department of Psychiatry, UMC Brain Center, University Medical Center Utrecht, Utrecht University, Utrecht, The Netherlands; 46 Department of Psychiatry, Seoul National University College of Medicine, Seoul, Republic of Korea; 47 Department of Brain and Cognitive Sciences, Seoul National University College of Natural Sciences, Seoul, Republic of Korea; 48 Department of Anatomy & Neurosciences, Amsterdam UMC, Location VUmc, Amsterdam Neuroscience, Amsterdam, The Netherlands; 49 Department of Sleep and Cognition, Netherlands Institute for Neuroscience, Amsterdam, The Netherlands; 50 Faculty of Computer Science, Dalhousie University, Halifax, NS Canada; 51 Child & Adolescent Psychiatry, University of California, Los Angeles, Los Angeles, CA USA; 52 Laboratory of Neuropsychiatry, IRCCS Santa Lucia Foundation, Rome, Italy; 53 Melbourne Neuropsychiatry Centre, Department of Psychiatry, The University of Melbourne, Melbourne, VIC Australia; 54 Orygen, The National Centre of Excellence in Youth Mental Health, Melbourne, VIC Australia; 55 CIBERSAM-G17, Madrid, Spain; 56 Department of Psychobiology and Methodology in Health Sciences, Universitat Autònoma de Barcelona, Barcelona, Spain; 57 Department of Psychiatry and Behavioral Sciences, Baylor College of Medicine, Houston, TX USA; 58 Department of Psychiatry and Biobehavioral Sciences, Semel Institute for Neuroscience and Human Behavior, University of California, Los Angeles, Los Angeles, CA USA; 59 Department of Mental Health, Veterans Affairs Greater Los Angeles Healthcare System, Los Angeles, CA USA; 60 Institute for Community Medicine, University Medicine Greifswald, Greifswald, Germany; 61 Stanley Center for Psychiatric Research, The Broad Institute, Cambridge, MA USA; 62 Analytic and Translational Genetics Unit, Massachusetts General Hospital, Boston, MA USA; 63 Psychiatry and Integrative Neurophysiology, VU University, Amsterdam UMC, Amsterdam, The Netherlands; 64 German Centre for Cardiovascular Research, Partner Site Greifswald, Greifswald, Germany; 65 Department of Psychology, University of Bath, Bath, UK; 66 Psychiatry and Behavioral Sciences, Northwestern University Feinberg School of Medicine, Chicago, IL USA; 67 Radiology, Northwestern University Feinberg School of Medicine, Chicago, IL USA; 68 Queensland Brain Institute, University of Queensland, Brisbane, QLD Australia; 69 Centre for Advanced Imaging, University of Queensland, Brisbane, QLD Australia; 70 Seoul National University Hospital, Seoul, Republic of Korea; 71 Yeongeon Student Support Center, Seoul National University College of Medicine, Seoul, Republic of Korea; 72 Department of Computer Science and Electrical Engineering, University of Maryland, Baltimore County, MD USA; 73 Department of Psychiatry and Behavioral Sciences, SUNY Upstate Medical University, Syracuse, NY USA; 74 Department of Psychiatry, University of Maryland School of Medicine, Baltimore, MD USA; 75 Norwegian Centre for Mental Disorders Research (NORMENT), Division of Mental Health & Addiction, Institute of Clinical Medicine, University of Oslo, Oslo, Norway; 76 Department of Clinical Neuroscience, Centre for Psychiatric Research, Karolinska Institutet, Stockholm, Sweden; 77 Department of Psychiatric Research, Diakonhjemmet Hospital, Oslo, Norway; 78 Department of Psychiatry, Amsterdam UMC, Location VUmc, Amsterdam Neuroscience, Amsterdam, The Netherlands; 79 Department of Research & Innovation, GGZ InGeest, Amsterdam, The Netherlands; 80 University of Groningen, University Medical Center Groningen, Groningen, The Netherlands; 81 Psychiatry, Pediatrics, and Psychological Sciences, University of Vermont, Burlington, VT USA; 82 Centre for Medical Image Computing (CMIC), Department of Medical Physics and Biomedical Engineering, University College London, London, UK; 83 Division of Mental Health and Addiction, Oslo University Hospital, Oslo, Norway; 84 Provost and Senior Vice President, Western University of Health Sciences, Pomona, CA USA; 85 Department of Radiology, Loma Linda University Medical Center, Loma Linda, CA USA; 86 Institute of Psychology, Leiden University, Leiden, The Netherlands; 87 Department of Psychiatry, Leiden University Medical Center, Leiden, The Netherlands; 88 Leiden Institute for Brain and Cognition, Leiden, The Netherlands; 89 Department of Psychology, Yale University, New Haven, CT USA; 90 Department of Psychology, University of California, Los Angeles, CA USA; 91 Department of Cognitive Neuroscience, Donders Institute for Brain, Cognition and Behaviour, Radboud University Medical Center, Nijmegen, The Netherlands; 92 Cognitive Neuroscience Unit, School of Psychology, Deakin University, Burwood, VIC Australia; 93 Department of Child and Adolescent Psychiatry/Psychology, Erasmus Medical Centre, Rotterdam, The Netherlands; 94 Department of Epidemiology, Erasmus Medical Centre, Rotterdam, The Netherlands; 95 Center for Cognitive Aging and Memory, University of Florida, Gainesville, FL USA; 96 Clinical and Health Psychology, Gainesville, FL USA; 97 Centre for Medical Image Computing (CMIC), Department of Computer Science, University College London, London, UK; 98 Dementia Research Centre, Institute of Neurology, University College London, London, UK; 99 Universite de Montreal, Centre de Recherche CHU Ste-Justine, Montreal, QC Canada; 100 School of Psychology and Centre for Human Brain Health, University of Birmingham, Birmingham, UK; 101 Department of Neurology, University of Utah, Salt Lake City, UT USA; 102 Psychiatry Neuroimaging Laboratory, Brigham & Women’s Hospital, Harvard Medical School, Boston, MA USA; 103 Social, Genetic & Developmental Psychiatry Centre, Institute of Psychiatry, Psychology & Neuroscience, King’s College London, London, UK; 104 Department of Psychology, School of Arts and Social Sciences, City, University of London, London, UK; 105 Department of Neuroimaging, Institute of Psychology, Psychiatry and Neurosciences, King’s College London, London, UK; 106 Division of Psychological and Social Medicine and Developmental Neurosciences, Faculty of Medicine, TU Dresden, Dresden, Germany; 107 Department of Rehabilitation and Movement Sciences, School of Health Professions, Rutgers Biomedical Health Sciences, Newark, NJ USA; 108 Department of Psychiatry and Mental Health, University of Cape Town, Cape Town, South Africa; 109 SU/UCT MRC Unit on Risk & Resilience in Mental Disorders, University of Stellenbosch, Stellenbosch, South Africa; 110 Department of Psychiatry, Icahn School of Medicine at Mount Sinai, New York, NY USA; 111 University of British Columbia, Vancouver, Canada; 112 Department of Psychiatry, Radboud University Medical Center, Nijmegen, The Netherlands; 113 Department of Psychiatry, University of Vermont, Burlington, VT USA; 114 Department of Psychiatry, Boston Children’s Hospital and Harvard Medical School, Boston, MA USA; 115 Olin Neuropsychiatric Research Center, Institute of Living, Hartford, CT USA; 116 Biomedical Engineering, Illinois Institute of Technology, Chicago, IL USA; 117 Institute for Information Transmission Problems, Kharkevich Institute, Moscow, Russian Federation; 118 Turner Institute for Brain and Mental Health & School of Psychological Sciences, Monash University, Melbourne, VIC Australia; 119 Department of Psychiatry & Neuropsychology, School for Mental Health and Neuroscience, Maastricht University, Maastricht, The Netherlands; 120 Genentech, Inc., South San Francisco, CA USA; 121 Department of Psychology, Penn State University, University Park, PA USA; 122 Social Life and Engineering Sciences Imaging Center, University Park, PA USA; 123 Department of Psychiatry, University of Pittsburgh, Pittsburgh, PA USA; 124 Research and Scientific Institute of Pediatrics and Child Health, CCH RAS, Ministry of Science and Higher Education, Moscow, Russian Federation; 125 Department of Pediatrics, Michigan State University, East Lansing, MI USA; 126 Institute for Quantitative Health Science and Engineering, East Lansing, MI USA; 127 Department of Psychiatry, University of North Carolina at Chapel Hill, Chapel Hill, NC USA; 128 CBRAIN, Department of Child and Adolescent Psychiatry, Psychosomatics, and Psychotherapy, Ludwig-Maximilians-Universität München, Munich, Germany; 129 Stevens Neuroimaging and Informatics Institute, Keck School of Medicine, University of Southern California, Los Angeles, CA USA; 130 Chan Division of Occupational Science and Occupational Therapy, Los Angeles, CA USA; 131 Center for Clinical Spectroscopy, Brigham and Women’s Hospital, Boston, MA USA; 132 Harvard Medical School, Boston, MA USA; 133 National Center for PTSD at Boston VA Healthcare System, Boston, MA USA; 134 Department of Psychiatry, Boston University School of Medicine, Boston, MA USA; 135 Biomedical Genetics, Boston University School of Medicine, Boston, MA USA; 136 School of Psychology, University of Auckland, Auckland, New Zealand; 137 Laboratory of Neuro Imaging, Mark and Mary Stevens Neuroimaging and Informatics Institute, Keck School of Medicine, University of Southern California, Los Angeles, CA USA; 138 Department of Psychiatry and Human Behavior, University of California, Irvine, Irvine, CA USA; 139 Mind Research Network, Albuquerque, NM USA; 140 Psychiatry, San Diego, CA USA; 141 The Kavli Foundation, Los Angeles, CA USA; 142 Department of Psychosis Studies, Institute of Psychiatry, Psychology & Neuroscience, King’s College London, London, UK; 143 Department of Psychiatry, Duke University School of Medicine, Durham, NC USA; 144 Mental Illness Research Education and Clinical Center, Durham VA Medical Center, Durham, NC USA; 145 Experimental Clinical & Health Psychology, Ghent University, Ghent, Belgium; 146 Department of Personality, Psychological Assessment and Treatment, University of Deusto, Bilbao, Spain; 147 Radiology and Biomedical Imaging, San Francisco, CA USA; 148 Department of Pediatrics, Russian National Research Medical University MoH RF, Moscow, Russian Federation; 149 Department of Psychology, Norwegian University of Science and Technology, Trondheim, Norway; 150 Department of Physical Medicine and Rehabilitation, St. Olavs Hospital, Trondheim University Hospital, Trondheim, Norway; 151 Biological Sciences, Purdue University, West Lafayette, IN USA; 152 National Institute of Mental Health Intramural Research Program, Bethesda, MD USA; 153 Department of Genetics and Computational Biology, QIMR Berghofer Medical Research Institute, Brisbane, QLD Australia; 154 Department of Neurodegenerative Disease, UCL Queen Square Institute of Neurology, London, UK; 155 Max Planck Institute of Psychiatry, Munich, Germany; 156 Centre for Youth Mental Health, The University of Melbourne, Melbourne, VIC Australia; 157 Department of Psychiatry and Psychotherapy, Charite, Humboldt University, Berlin, Germany; 158 Department of Radiology, Keck School of Medicine of USC, University of Southern California, Los Angeles, CA USA; 159 Department of Clinical and Experimental Epilepsy, University College London, London, UK; 160 Chalfont Centre for Epilepsy, Chalfont St Peter, UK; 161 Department of Medical Genetics, Oslo University Hospital, Oslo, Norway; 162 Department of Psychiatry & Neuroscience Institute, SA MRC Unit on Risk & Resilience in Mental Disorders, Cape Town, South Africa; 163 Department of Genetics & UNC Neuroscience Center, University of North Carolina at Chapel Hill, Chapel Hill, NC USA; 164 Institute of Medical Science and Technology, Shahid Beheshti University, Tehran, I. R. Iran; 165 Department of Neurology, TBI and Concussion Center, Salt Lake City, UT USA; 166 Missouri Institute of Mental Health, Berkeley, MO USA; 167 Psychology Department & Neuroscience Institute, Georgia State University, Atlanta, GA USA; 168 Clinical Translational Neuroscience Laboratory, Department of Psychiatry and Human Behavior, University of California Irvine, Irvine, CA USA; 169 Center for the Neurobiology of Learning and Memory, University of California, Irvine, Irvine, CA USA; 170 Donders Centre for Cognitive Neuroimaging, Radboud University Medical Centre, Nijmegen, The Netherlands; 171 Division of Mind and Brain Research, Department of Psychiatry and Psychotherapy CCM, Charité - Universitätsmedizin Berlin, corporate member of Freie Universität Berlin, Humboldt-Universität zu Berlin, and Berlin Institute of Health, Berlin, Germany; 172 Molecular and Cellular Therapeutics, Royal College of Surgeons in Ireland, Dublin, Ireland; 173 Research and Early Development, Biogen Inc, Cambridge, MA USA; 174 VA Salt Lake City Healthcare System, Salt Lake City, UT USA; 175 Department of Physical Medicine and Rehabilitation, Baylor College of Medicine, Houston, TX USA; 176 Keck School of Medicine, University of Southern California, Los Angeles, CA USA; 177 Skolkovo Institute of Science and Technology, Moscow, Russian Federation

**Keywords:** Biomarkers, Neuroscience, Scientific community, Genomics, Psychiatric disorders

## Abstract

This review summarizes the last decade of work by the ENIGMA (Enhancing NeuroImaging Genetics through Meta Analysis) Consortium, a global alliance of over 1400 scientists across 43 countries, studying the human brain in health and disease. Building on large-scale genetic studies that discovered the first robustly replicated genetic loci associated with brain metrics, ENIGMA has diversified into over 50 working groups (WGs), pooling worldwide data and expertise to answer fundamental questions in neuroscience, psychiatry, neurology, and genetics. Most ENIGMA WGs focus on specific psychiatric and neurological conditions, other WGs study normal variation due to sex and gender differences, or development and aging; still other WGs develop methodological pipelines and tools to facilitate harmonized analyses of “big data” (i.e., genetic and epigenetic data, multimodal MRI, and electroencephalography data). These international efforts have yielded the largest neuroimaging studies to date in schizophrenia, bipolar disorder, major depressive disorder, post-traumatic stress disorder, substance use disorders, obsessive-compulsive disorder, attention-deficit/hyperactivity disorder, autism spectrum disorders, epilepsy, and 22q11.2 deletion syndrome. More recent ENIGMA WGs have formed to study anxiety disorders, suicidal thoughts and behavior, sleep and insomnia, eating disorders, irritability, brain injury, antisocial personality and conduct disorder, and dissociative identity disorder. Here, we summarize the first decade of ENIGMA’s activities and ongoing projects, and describe the successes and challenges encountered along the way. We highlight the advantages of collaborative large-scale coordinated data analyses for testing reproducibility and robustness of findings, offering the opportunity to identify brain systems involved in clinical syndromes across diverse samples and associated genetic, environmental, demographic, cognitive, and psychosocial factors.

## Introduction

**Fig. 1:**
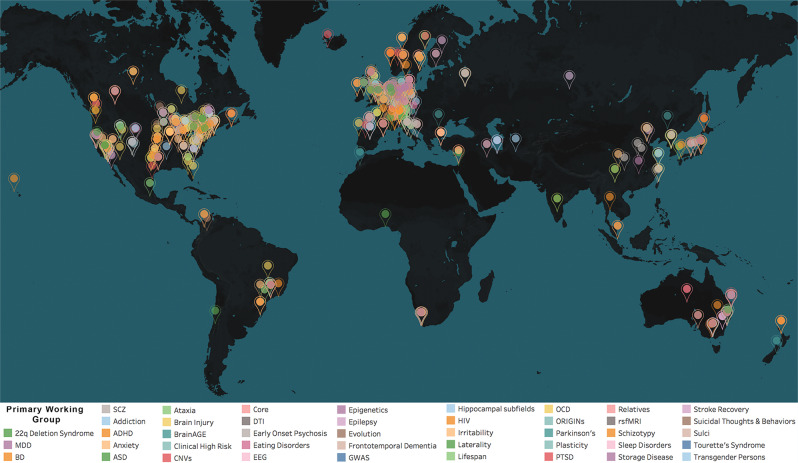
World Map of ENIGMA’s Working Groups. The ENIGMA Consortium has grown to include over 1400 participating scientists from over 200 institutions, across 43 countries worldwide. ENIGMA is organized as a set of 50 WGs, studying 26 major brain diseases (see color key). Each group works closely with the others and consists of worldwide teams of experts in each brain disorder as well as experts in the major methods used to study each disorder. The diseases studied include major depressive disorder, bipolar disorder, schizophrenia, substance use disorder, post-traumatic stress disorder, attention-deficit/hyperactivity disorder, obsessive-compulsive disorder, and autism spectrum disorder, and several neurological disorders, including Parkinson’s disease, epilepsy, ataxia, and stroke. In recent years, new WGs were created that grew into worldwide consortia on epilepsy (Whelan et al.^9^), eating disorders (King et al.^104^), anxiety disorders (Groenewold et al.^107^), antisocial behavior, and infant neuroimaging.

The ENIGMA (Enhancing NeuroImaging Genetics through Meta Analysis) Consortium is a collaboration of more than 1400 scientists from 43 countries studying the human brain. ENIGMA started 10 years ago, in 2009, with the initial aim of performing a large-scale neuroimaging genetic study, and has since diversified into 50 working groups (WGs), pooling worldwide data, resources and expertise to answer fundamental questions in neuroscience, psychiatry, neurology, and genetics (Fig. 1 shows a world map of participating sites, broken down by working group). Thirty of the ENIGMA WGs focus on specific psychiatric and neurologic conditions. Four study different aspects of development and aging. Others study key transdiagnostic constructs, such as irritability, and the importance of evolutionarily interesting genomic regions in shaping human brain structure and function. Central to the success of these WGs are the efforts of dedicated methods development groups within ENIGMA. There are currently 12 WGs that develop and disseminate multiscale and ‘big data’ analysis pipelines to facilitate harmonized analyses using genetic and epigenetic data, multimodal (anatomical, diffusion, functional) magnetic resonance imaging (MRI) and spectroscopy (MRS) measures, in combination with genetic and epigenetic data, and data from electroencephalography (EEG).

The Consortium has been a formidable force for discovery and innovation in human brain imaging, supporting more than 200 active studies. The disorder-specific WGs have published the largest neuroimaging studies to date in schizophrenia (SCZ; total *N* = 9572; 4474 cases)^1^, bipolar disorder (BD; total *N* = 6503; 2447 cases)^2^, major depressive disorder (MDD; total *N* = 10,105; 2148 cases)^3^, post-traumatic stress disorder (PTSD; total *N* = 1868; 794 cases)^4^, substance use disorders (SUD; total *N* = 3240; 2140 cases)^5^, obsessive-compulsive disorder (OCD; total *N* = 3665; 1905 cases)^6^, attention-deficit/hyperactivity disorder (ADHD; total *N* = 4180; 2246 cases)^7^, autism spectrum disorders (ASD; total *N* = 3222; 1571 cases)^8^, epilepsy (*N* = total 3876; 2149 cases)^9^, and 22q11.2 deletion syndrome (22q11DS; total *N* = 944; 474 cases)^10^. Key results of these studies are summarized in Table 1. Building on this work, the focus of the ENIGMA disorder-specific WGs now goes beyond traditional diagnostic boundaries. As these first large-scale studies are being completed, ENIGMA is beginning to identify shared and distinct neuroimaging patterns in brain disorders with known genetic or clinical overlap^11,12^, and to delineate the role of transdiagnostic risk factors (e.g., childhood trauma) and clinical phenomena (e.g., suicidal thoughts and behaviors). In addition, ENIGMA’s genetic studies are now analyzing imaging and genetics data from more than 50,000 people to uncover genetic markers that most robustly associated with brain structure and function, or imaging derived neurobiological traits related to various disease conditions^13–16^.

**Table 1:** A Selection of key findings from ENIGMA’s Working Groups, along with key papers and current sample sizes.

Working group	Number of datasets	Total *N* (patient *N*)	Age range (in years)	Relevant publications	Main findings
*Clinical*					
22Q11DS	14	863 (533)	6–56	Villalón-Reina et al.^17^; Sun et al.^10^	Widespread reductions in diffusivity, pronounced in regions with major cortico-cortical and cortico-thalamic fibers; thicker cortical gray matter overall, but focal thickness reduction in temporal and cingulate cortex; cortical surface area showed pervasive reductions; lower cortical surface area in individuals with larger microdeletion; 22q-related psychosis associated with lower cortical thickness and significantly overlapped with findings from ENIGMA-SCZ group.
Addiction/SUDs	118	18,823 (6,592)	7–68	Mackey et al.^5,84^; Conrod et al.^86^	Common neural substrate shared in dependence; differential patterns of regional volume as biomarkers of dependence on alcohol and nicotine; lower volume or thickness observed, with greatest effects associated with alcohol use disorder; insula and medial orbitofrontal cortex affected, regardless of dependence.
ADHD	37	4180 (2246)	4–63	Hoogman et al.^7,91^; Klein et al.^47^; Zhang-James^94^; Hess et al.^92^	Reduction in bilateral amygdala, striatal, and hippocampal volumes in the ADHD population, especially in children; lower cortical surface area values found in children with ADHD, but not in adolescents or adults; lower surface area associated with ADHD symptoms in the general population in childhood; genetic association studies suggest that genes involved in neurite outgrowth play a role in findings of reduced volume in ADHD; gene-expression studies imply that structural brain alterations in ADHD can also be explained in part by the differential vulnerability of these regions to mechanisms mediating apoptosis, oxidative stress, and autophagy.
ASD	54	3583 (1774)	2–64	Postema et al.^97^; van Rooij et al.^8^	Altered morphometry in the cognitive and affective parts of the striatum, frontal cortex and temporal cortex in ASD.
BD	44	11,100 (3100)	8–86	Favre et al.^69^; Nunes et al.^23^; Hibar et al.^2,68^	Volumetric reductions in hippocampus and thalamus and enlarged lateral ventricles in patients; thinner cortical gray matter in bilateral frontal, temporal and parietal regions; strongest effects on left pars opercularis, fusiform gyrus and rostral middle frontal cortex in BD.
Eating Disorders	28 anorexia nervosa (AN); 12 bulimia nervosa (BN)	2531 (897 AN; 307 BN)	10–50 AN; 12–46 BN	Walton et al.^48^	Signs of inverse concordance between greater thalamus volume and risk for anorexia nervosa (AN); variation in gene DRD2 significantly associated with AN only after conditioning on its association with caudate volume; genetic variant linked to LRRC4C reached significance after conditioning on hippocampal volume.
Epilepsy	24	3876 (2149)	18–55	Whelan et al.^9^	Patients with IGE showed volume reductions in the right thalamus and lower thickness in the bilateral precentral gyri; both MTLE subgroups showed volume reductions in the ipsilateral hippocampus, and lower thickness in extrahippocampal cortical regions, including the precentral and paracentral gyri; lower subcortical volume and cortical thickness were associated with a longer duration of epilepsy in the all-epilepsies and right MTLE groups.
HIV	12	1044 (all patients)	22–81	Nir et al.^124,169,170^; Fouche et al.^171^	In the full group, subcortical volume associations implicated the limbic system: lower current CD4+ counts were associated with smaller hippocampal and thalamic volumes; a detectable viral load was associated with smaller hippocampal and amygdala volumes; limbic effects were largely driven by participants on cART; in subset of participants not on cART, smaller putamen volumes were associated with lower CD4+ count.
MDD	38	14,249 (4379)	10–89	van Velzen et al.^67^; Tozzi et al.^75^; Han et al.^72^; Frodl et al.^74^; Renteria et al.^172^; Schmaal et al.^3,70^; Ho et al.^137^; Saemann et al.^83^	Significantly lower hippocampal volumes; thinner orbitofrontal cortex, anterior and posterior cingulate, insula and temporal lobes cortex in adult MDD patients; lower total surface area and regional reductions in frontal regions and primary and higher-order visual, somatosensory and motor areas in adoloescent MDD patients; greater exposure to childhood adversity associated with smaller caudate volumes in females, independent of MDD; patients reporting suicidal plans or attempts showed a smaller ICV volume compared to controls.
OCD	38	3665 (1905)	5–65	Boedhoe et al.^6,88,167^; Hibar et al.^45^	Subcortical abnormalities in pediatric and adult patients; pallidum (bigger) and hippocampus (smaller) key in adults, and thalamus (bigger) key in (unmedicated) pediatric group; parietal cortex consistently implicated both in children and adults; more widespread cortical thickness abnormalities in medicated adults, and more pronounced surface area deficits (mainly in frontal regions) in medicated pediatric OCD patients.
PTSD	16	3118 (1288)	17–85	Dennis et al.^76^; Salminen et al.^80^; Logue et al.^4^; O’Leary et al.^78^	Significantly smaller hippocampi, on average, in individuals with current PTSD compared with trauma-exposed control subjects, and smaller amygdalae.
Schizophrenia	39	9572 (4474)	18–77	Holleran et al.^57^; van Erp et al.^1,54,55^; Kelly et al.^56^; Walton et al.^62,63^; Kochunov et al.^66^	Positive symptom severity was negatively related to bilateral STG thickness; widespread thinner cortex and smaller surface area, largest effect sizes in frontal and temporal lobe regions; smaller hippocampus, amygdala, thalamus, accumbens and intracranial volumes; larger pallidum and lateral ventricle volumes; widespread reductions in FA, esp. in anterior corona radiata and corpus callosum; higher mean and radial diffusivity; left MOFC thickness significantly associated with negative symptom severity; link between prefrontal thinning and negative symptom severity in schizophrenia.
CNV	37	16,889 (24 16p11.2 distal and 125 15q11.2 CNV carriers)	3–90	van der Meer et al.^100^; Sonderby^53^	16p11.2 distal CNV: Negative dose-response associations with copy number on intracranial volume and regional caudate, pallidum and putamen volumes. 15q11.2 CNV: Decrease in accumbens and cortical surface area in deletion carriers and negative dose response on cortical thickness.
*Non-clinical*					
EEG	5	8425	5–73	Smit et al.^40^	Identified several novel genetic variants associated with oscillatory brain activity; replicated and advanced understanding of previously known genes associated with psychopathology (i.e., schizophrenia and alcohol use disorders); these psychopathological liability genes affect brain functioning, linking the genes’ expression to specific cortical/subcortical brain regions.
GWAS	34	22,456	3–91	Satizabal et al.^14^; Grasby et al.^13^; Hibar et al.^25,173^; Adams et al.^169^	Over 200 genetic loci where common variation is associated with cortical thickness or surface area; over 40 common genetic variants associated with subcortical volumes.
Laterality	99	17,141	3–90	de Kovel et al.^71^; Kong et al.^90,154^; Postema et al.^97^; Guadalupe et al.^174^	Average patterns of left-right anatomical asymmetry of the healthy brain were mapped, as regards cortical regional surface areas, thicknesses, and subcortical volumes; fronto-occipital gradient in cortical thickness asymmetry was found, with frontal regions generally thicker on the left, and occipital regions on the right; asymmetries of various structural measures were significantly heritable, indicating genetic effects that differ between the two sides; age, sex and intracranial volume affected some asymmetries, but handedness did not; disorder case–control analyses revealed subtle reductions of regional cortical thickness asymmetries in ASD, as well as altered orbitofrontal surface area asymmetry; little evidence for altered anatomical asymmetry was found in MDD; pediatric patients with OCD showed evidence for altered asymmetry of the thalamus and pallidum.
Lifespan	91	14,904 healthy individuals	2–92	Dima et al.^175^; Frangou et al.^176^	Thickness in almost all cortical regions decreased prominently in the first two to three decades of life, with an attenuated or plateaued slope afterwards; exceptions to this pattern were entorhinal and temporopolar cortices whose thickness showed an attenuated inverse U-shaped relation with age, and anterior cingulate cortex, which showed a U-shaped association with age; age at peak cortical thickness was 6–7 years for most brain regions.
Plasticity	36	10,199 (2242)	6–97	Brouwer et al.^38,39^	Heritability estimates of change rates were generally higher in adults than in children suggesting an increasing influence of genetic factors explaining individual differences in brain structural changes with age; for some structures, the genetic factors influencing change were different from those influencing the volume itself, suggesting the existence of genetic variants specific for brain plasticity.

As we detail in this review, the ENIGMA Consortium has made multiple, seminal contributions to neuroscience and psychiatry, including (a) characterization of robust neuroimaging profiles for various brain disorders, (b) standardization of metrics used to assess clinical symptoms of patients across multiple research sites, and (c) use of dimensional approaches that go beyond the case–control comparisons of individuals with categorical diagnoses, and further enable the investigation of specific genetic, and environmental features or neurobiological markers associated with disorder risk and treatment outcome. The large scale and inclusivity of these analyses—in terms of populations, sample sizes, numbers of coordinating centers, and diversity of imaging and genetic data—has been instrumental for demonstrating robust associations between clinical factors and brain alterations, and for stratifying patients with the same diagnosis according to differential treatment outcomes^10,17^. Thus, a valuable aspect of the existing ENIGMA studies is the ability to identify the most robust pattern of non-invasively measured neurobiological features involved in clinical syndromes across multiple samples that are more representative of the global population. This also results in robust effect size estimates, without the confounds of literature-based meta-analyses based on published data with possible publication bias (as noted in Kong et al.)^18^. These data also provide a unique opportunity to assess important sources of disease heterogeneity, including key genetic, environmental, demographic, and psychosocial factors. Here, we provide a synopsis of the first decade of ENIGMA’s activities and highlight the successes and challenges encountered along the way.

## History

ENIGMA was launched in December 2009 to help ‘break the logjam’ in genetic studies of the brain. At the time, most neuroimaging genetics studies were assessing historically candidate genetic variations, mostly in very small samples of a few tens to hundreds of participants (e.g., *COMT*, *5-HTTLPR, BDNF*). These studies typically reported ‘candidate gene’ effects that did not replicate when tested in independent cohorts^19–21^. It became apparent that very large numbers of genetic loci contributed to variation in complex neurological or psychiatric traits, including imaging-derived brain measures—each with a very small effect size—and only a few genetic loci accounted for more than 1% of the variance in any complex brain condition or measure^22^. Thus, scientists began to recognize the need to pool multiple datasets worldwide to perform better-powered studies of these traits. In response, the ENIGMA Consortium’s initial plan was to merge two ‘big data’ sources—neuroimaging and genetics—with the aim of discovering the impact of genetic factors on brain systems, to determine whether these genetic factors underlie manifestation of disorders within the brain, and to identify diagnostic and prognostic neuroimaging biomarkers. A further goal was to improve on previous literature-based meta-analyses by using harmonized processing and analysis protocols on an unprecedented scale. This was the impetus that launched ENIGMA’s early studies.

**Fig. 2:**
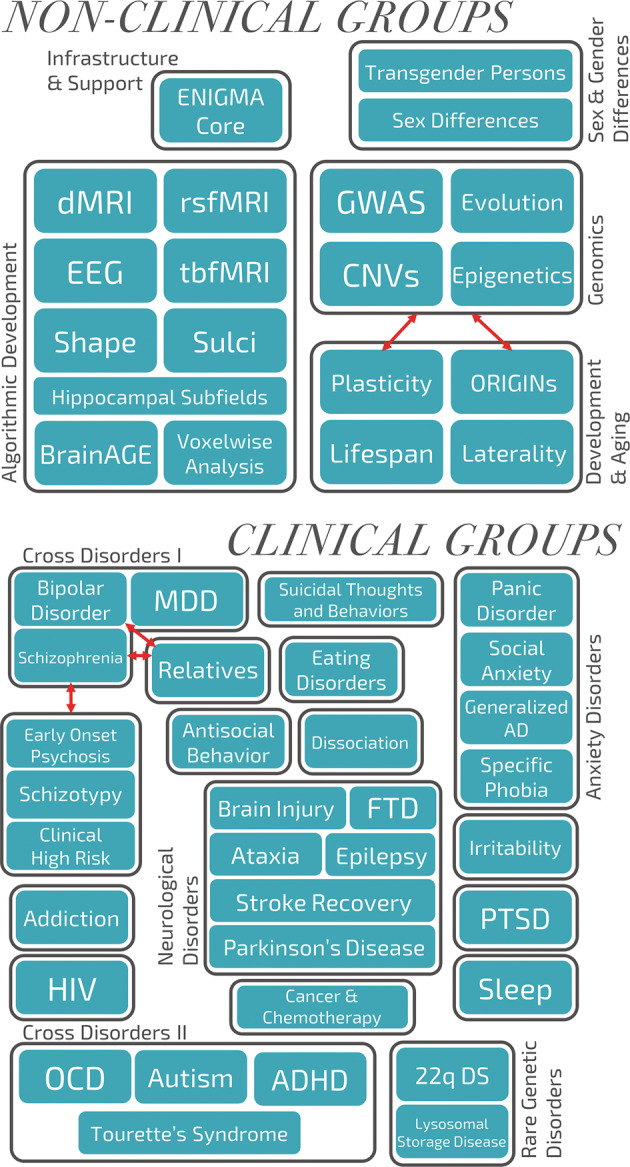
ENIGMA’s Working Group Flowchart. ENIGMA’s working groups are divided into technical groups that work on testing harmonized methods, and clinical groups that study different disorders and conditions across psychiatry and neurology, as well as some behaviors (e.g., schizotypy and antisocial behaviors). The use of harmonized analysis methods across all the working groups has enabled cross-disorder comparisons (e.g., in the affective/psychosis spectrum of depression to bipolar disorder to schizophrenia), and transdiagnostic analyses of risk factors such as childhood trauma across a number of disorders (such as major depressive disorder (MDD) and post-traumatic stress disorder (PTSD)). Several working groups, such as brain trauma and anxiety, consist of several subgroups examining subtypes (e.g., panic disorder or social anxiety), and allow analyses of overlap and differences (e.g., between military and civilian brain trauma).

In 2014, the NIH Big Data to Knowledge (BD2K) program awarded a consortium grant to ENIGMA with seed funding for WGs on nine disorders: SCZ, BD, MDD, OCD, ADHD, ASD, SUD, 22q11DS, and the effects of the human immunodeficiency virus (HIV) on the brain. This support led to the largest neuroimaging studies for the nine targeted disorders, with results reported in over 50 manuscripts. These initial successes provided the driving force to establish an additional 21 disease WGs (see Working Group chart, Fig. 2).

Following the model established by the Psychiatric Genomics Consortium (PGC), which emphasized harmonization of genomic analysis protocols across sites, the ENIGMA Consortium created harmonized protocols to analyze brain structure and function, along with genetic, and clinical data across its WGs. Instead of centralizing data, ENIGMA opted to work as a ‘distributed consortium’, asking groups to run standardized protocols themselves, rather than the approach used in the PGC, where data are centralized. At the time, ENIGMA design was important for the rapid acceptance of the consortium in the field, as it made contribution very easy; further, the memoranda of understanding provided the basic guidelines for the trusted collaborative networks to develop. In the meantime—with views on data sharing having changed quite considerably—many ENIGMA WGs now also share (derived) individual data, allowing for more in-depth analyses.

In ENIGMA’s genetic studies, many participating centers use different genotyping chips, so data were first imputed to common genomic references (such as the 1000 Genomes reference panel), allowing each participating site to perform the same association tests between brain measures and genetic variation at over 10 million loci across the genome. Furthermore, the ENIGMA Consortium standardized procedures for the extraction of brain metrics (such as cortical thickness, cortical surface area, and subcortical volume) from raw neuroimaging data, implemented consensus protocols for data quality control and outlier handling, and pioneered new meta-analytic methods for the analysis of aggregated statistical information (http://enigma.ini.usc.edu/protocols/). ENIGMA’s meta-analyses estimated the size and precision of the effects after pooling evidence from multiple cohorts, and they also ranked the neuroimaging effect sizes of findings emerging from case–control comparisons, thereby setting the stage for deeper, secondary analyses aiming to explore potential moderators of psychiatric and neurological disease. More recently, many ENIGMA groups have moved beyond cohort level meta-analyses to pooled, or ‘mega’-analyses (Using brain volumetric data from ENIGMA’s OCD, ADHD, and ASD working groups, Boedhoe et al.^12^ compared meta-analysis to mega-analyses that model site or cohort effects as random effects, showing broad agreement. Mega-analyses allow more sophisticated statistical adjustments as they pool more information across cohorts; meta-analyses tend to be more efficient when ethical, legal or logistic constraints govern or restrict individual-level data transfer (e.g., genome-wide genetic data).), where anonymized and unidentifiable individual-level data are aggregated in a central location, allowing more flexible statistical designs, such as machine learning analyses^23^, reliable estimation of interaction effects, and examination of polygenic risk scores. The type and amount of data transferred for each analysis is chosen pragmatically for each study. Distributed analyses promote scientific engagement from many groups worldwide and take advantage of distributed computing resources that scale up as the network grows; here the data transferred is mainly aggregate measures such as quality control metrics and the statistical metrics derived from agreed-upon analytical tests. On the other hand, the centralized analyses are preferable when a variable of interest is sparsely distributed across sites, (e.g., individuals with 22q11DS exhibiting psychotic symptoms) or when a specific method is being developed, and computational power or expertise is available at only a few sites; here the data transferred usually include unidentifiable derived imaging metrics (e.g., hippocampal volume) and demographic or clinical information (age at scan, sex, diagnostic status, etc.); however, this form of analysis may limit participation and requires individual data transfer agreements with participating sites. We note, because of these required agreements with potentially clinically sensitive patient information, and the project-specific design of the ‘centralized’ approaches, ENIGMA does not curate a database for repeated or open access, and each cohort PI approves of each project for which they contribute data.

## ENIGMA’s genetic studies

### Uncovering the genetic basis of brain morphometric variation

**Fig. 3:**
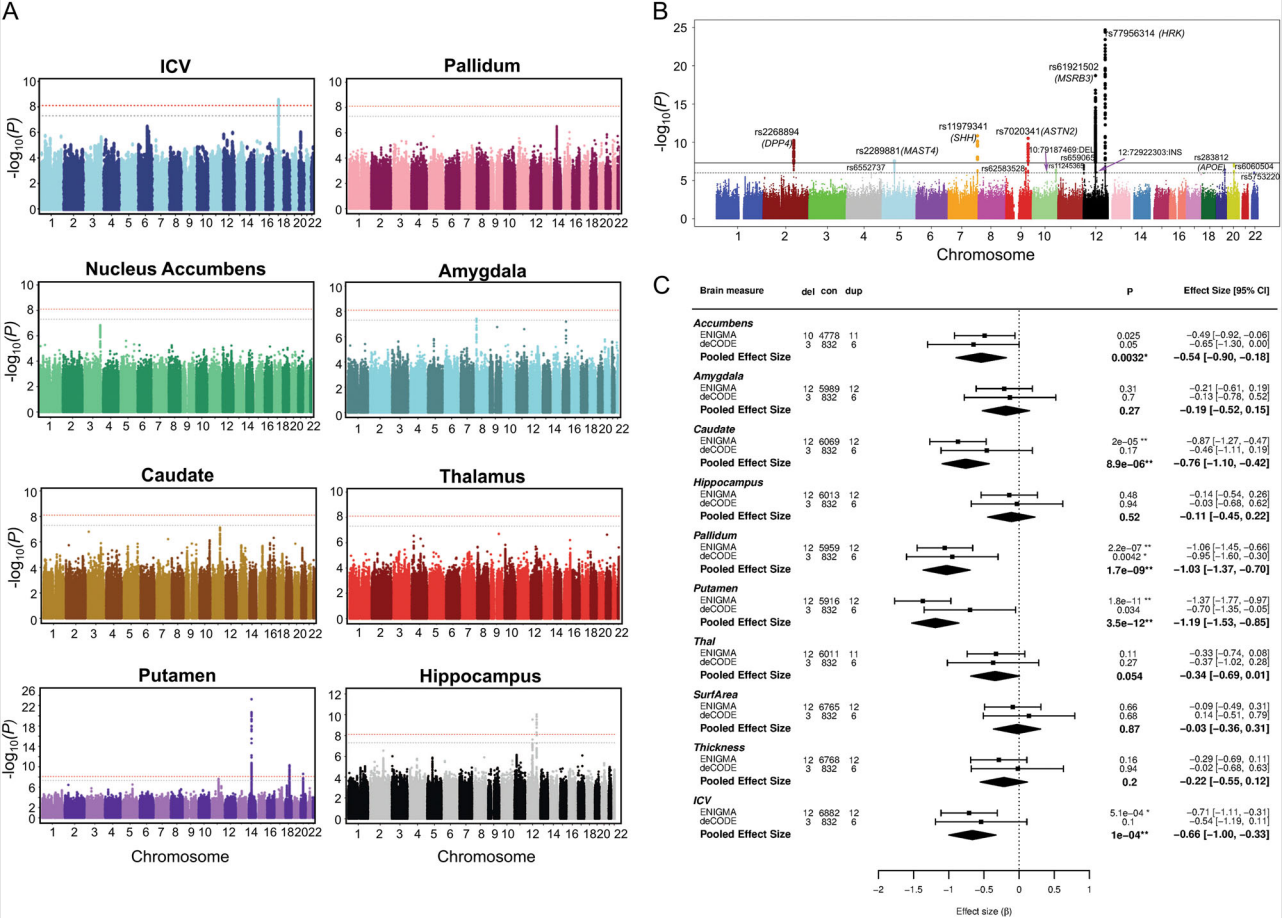
Genetic Influences on brain structure: effects of common and rare genetic variants. ENIGMA’s large-scale genetic analyses study the effects of both common and rare genetic variants on brain measures. **a** A series of progressively larger genome-wide association studies have revealed over 45 genetic loci associated with subcortical structure volumes (Hibar et al.^25^, Satizabal et al.^14^) and over 200 genetic loci associated with cortical thickness and surface area Grasby et al.^13^. The Manhattan plots here (adapted from Hibar et al.^25^, show the genome (on the *x*-axis) and the evidence for association (as a logarithm of the *p*-value, on the *y*-axis) for each common genetic variant (or SNP) with the volume of each brain structure shown. **b** Genetics of Hippocampal Volume. A subsequent genome-wide association study (GWAS) of 33,536 individuals discovered six independent loci significantly associated with hippocampal volume, four of them novel. Of the novel loci, two lie within key genes involved in neuronal migration and microtubule assembly (*ASTN2* and *MAST4*) (Hibar et al.^173^). An interactive browser, ENIGMA-Vis—http://enigma-brain.org/enigmavis—can be used to navigate ENIGMA’s genomic data. Initially started as a web page to plot ENIGMA summary statistics data for a specific genomic region, ENIGMA-Vis grew over the years into a portal with tools to query, visualize, and navigate the effects, and relate them to other GWAS. **c** In complementary work on rare variants by the ENIGMA-CNV Working Group, Sønderby and colleagues (2018) examined effects of the 16p11.2 distal CNV that predisposes to psychiatric conditions including autism spectrum disorder and schizophrenia. ENIGMA (including the 16p11.2 European Consortium) and deCODE datasets were combined to discover negative dose-response associations with copy number on intracranial volume and regional caudate, pallidum and putamen volumes—suggesting a neuropathological pattern that may underlie the neurodevelopmental syndromes. The agreement across datasets is apparent in the Forest plots for each brain region. [Data adapted, with permission from the authors and publishers].

The first demonstration of the value of the ENIGMA approach was the identification of genetic loci associated with variation in subcortical volumes including the caudate, putamen, and hippocampus (see Fig. 3)^14,24,25^. These genome-wide association studies (GWAS) yielded intriguing new leads regarding the genetic architecture of the human brain that were only possible because ENIGMA afforded increased power to detect subtle effects. More recently, ENIGMA identified more than 200 individual loci that significantly contribute to variation in brain measures, with *p*-values reaching 10^−180^; each single locus accounted for only 0.1–1% of phenotypic variance, but up to 20% of the variance in aggregate. For this effort ENIGMA had partnered with the CHARGE Consortium and UK Biobank on a series of studies of 70 cortical measures, including regional cortical thickness and surface area^13^. These discoveries resulted in an annotated atlas of common genetic variants that contribute to shaping the human cerebral cortex. Of particular interest, we found that genetic loci affecting brain morphology show enrichment for developmentally regulated genes^13^ and human-specific regulatory elements^26,27^. Ongoing efforts are beginning to map these genetic effects at a finer-grained spatial resolution using shape analysis, surface- and voxel-based analyses^28–31^. Moving beyond the mass univariate methods, which analyze each brain measure separately, ENIGMA has begun to use multivariate methods to meet the challenge of quantifying the complex relationships between brain networks—or ‘connectomes’—and the genome^32–34^.

Current ENIGMA sample sizes (which now exceed 50,000) are sufficiently large to identify genetic associations at a pace comparable to that of GWAS for other phenotypes. In a recent analysis, Holland^35^ contrasted rates of discovery of genetic loci by ENIGMA and the PGC and noted the distribution of effect sizes for some brain measures (e.g., putamen volume) may indeed be enriched for slightly larger effects compared to behavioral traits (see also Le and Stein^36^ and Franke et al.^37^). Still, a central understanding gained from the ENIGMA association screens is that neuroimaging genetics studies—just like analyses of behavioral measures, require tens (perhaps hundreds) of thousands of participants to obtain robust and reproducible effects of common polymorphisms. Most individual effect sizes are very small explaining <0.2% of variance, as for other complex human traits. GWAS of multiple imaging measures may offer a way to parcellate the brain into clusters or sectors with overlapping genetic drivers, perhaps boosting the power to discover genetic loci, by aggregating regions based on their genetic correlation.

### Uncovering the genetic basis of brain change

The quest to discover genetic loci that modulate brain development and aging led to the launch of the ENIGMA-Plasticity WG^38^, which uses longitudinal brain imaging data from 36 cohorts worldwide to estimate rates of brain growth or atrophy, and performs GWAS to find genetic markers that may influence these rates of change. The ENIGMA-Plasticity WG has established the heritability of brain changes over time and has shown that distinct genetic factors influence regional brain volumes and their rate of change, implying the existence of genetic variants specifically associated with change^39^. The WG is further investigating how closely developmental and aging-related genes overlap, and how they overlap with genetic loci that are associated with risk for development of psychiatric and neurological disease throughout life. Overall, the high rate of discovery driven by ENIGMA is offering initial glimpses of the overlap among genetic drivers of brain change throughout life with specific markers of brain structure and function.

### Uncovering the genetic basis of brain functional variation

The ENIGMA Consortium has also carried out genetic association studies of EEG-derived phenotypes. The first study^40^ of the EEG WG performed the largest GWAS to date of oscillatory power across a range of frequencies (delta 1–3.75 Hz, theta 4–7.75 Hz, alpha 8–12.75 Hz, and beta 13–30 Hz) in 8425 healthy subjects. They identified several novel genetic variants associated with alpha oscillatory brain activity that were previously linked to psychiatric disorders.

### Characterizing the association between brain morphology and disease-risk genes

In an early ENIGMA study, minimal overlap was detected between schizophrenia-related and brain-related genetic loci^37^. These questions were revisited with Bayesian models^41^ and LD-score regression methods^42^ which identified stronger overlap between genetic loci involved in cortical structure and loci implicated in insomnia, major depression, Parkinson’s disease, and general cognitive ability or IQ^13^. Despite initial negative results^37^, ENIGMA’s growing sample size led to more powerful results, allowing for the recent successes in the discovery of brain-related genetic variants that also affect risk for schizophrenia^43,44^, OCD^45^, anxiety disorders^46^, PTSD^46^, ADHD^47^, anorexia nervosa^48^, Tourette syndrome^49^, and insomnia^13^.

As the sample size of brain scans in the ENIGMA Consortium increased beyond 50,000 MRI scans, it became possible to discover further genetic loci associated with multiple brain traits implicated in brain disorders. A recent example is an ENIGMA-CHARGE GWAS of white matter (WM) hyperintensities, a sign of vascular brain disease, by Mather et al. (in prep), which found heterogeneous effects for variants associated with lesions near the ventricles versus lesions elsewhere in the brain. An innovative feature of this analysis was the use of anatomical clustering of traits to yield more powerful brain GWAS results. Anatomical or genetic clustering is yet another methodological improvement implemented by ENIGMA, that can be used widely to enhance detection of genetic associations in multiple brain disorders (see Lorenzi, Couvy-Duchesne for other multivariate imaging GWAS approaches^50,51^).

### Uncovering the epigenetic basis of brain morphometric variation

Inspired by these successes, ENIGMA widened the scope of its WGs to embrace the study of epigenetic variations. ENIGMA’s Epigenetics group has already identified two sites in the genome where methylation relates to hippocampal volume (*N* = 3337)^52^. Ongoing studies focus on brain measures sensitive to epigenetic age, an index of biological as opposed to chronological aging, in both health and disease.

### From common nucleotide variations to rare copy number variants (CNV)

The ENIGMA-CNV WG was launched to study the effects of CNVs, relatively rare genetic variants predisposing individuals to various neuropsychiatric disorders. The ENIGMA collaborative approach is ideal for studying low-frequency variants, as such efforts require large samples that are usually beyond the scope of a single study. Their first reports were on the 16p11.2 distal^53^ and 15q11.295 CNVs (Fig. 3) and additional studies on other CNVs are underway.

### ENIGMA disorder-based neuroimaging studies

#### ENIGMA-schizophrenia

The Schizophrenia WG was formed in 2012, and has since analyzed data from 39 cohorts worldwide and has identified case–control differences in brain morphometry^1,54,55^ and WM microstructure^56,57^, on an unprecedented scale. ENIGMA-Schizophrenia was the first working group to publish large-scale analyses of disease, in two seminal papers on case–control differences in brain morphometry based on the largest samples to date. Van Erp and ENIGMA colleagues^54^ first reported that patients with SCZ (*N* = 2028 patients) had smaller hippocampus (Cohen’s *d* = −0.46), amygdala (*d* = −0.31), thalamus (*d* = −0.31), nucleus accumbens (*d* = −0.25), total intracranial volumes (*d* = −0.12), and larger pallidum (*d* = 0.21) and lateral ventricle volumes (*d* = 0.37) compared to healthy controls (*N* = 2540). In a subsequent study, the team expanded their sample to include 4474 individuals with SCZ and 5098 controls to study cortical structures^1^. Compared to healthy controls, patients with SCZ had globally thinner cortices (left/right hemisphere: *d* = −0.53/−0.52) and smaller overall cortical surface area (left/right hemisphere: *d* = −0.25/−0.25), with greatest effect sizes in frontal and temporal regions.

**Fig. 4:**
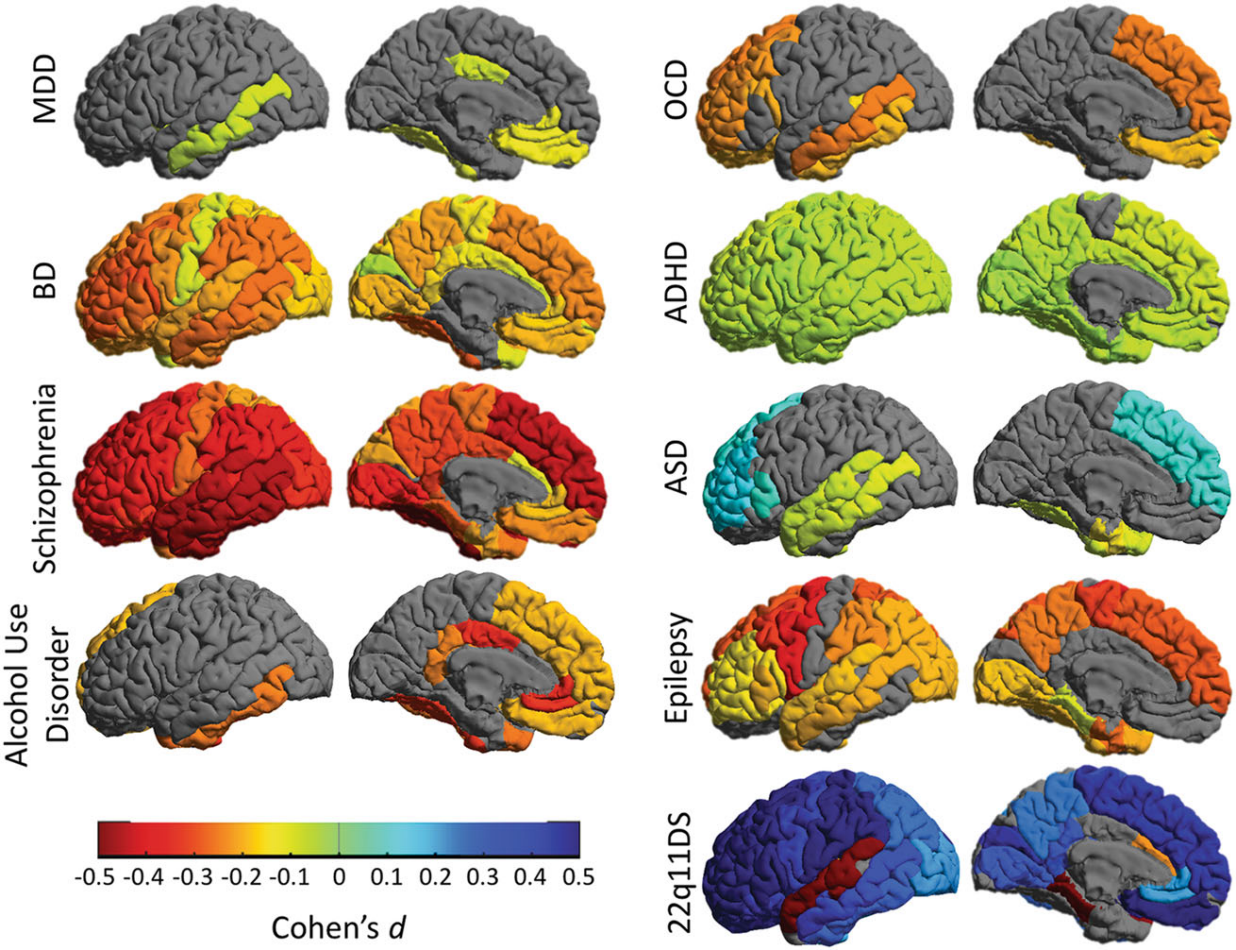
ENIGMA’s large-scale studies of nine brain disorders. Cortical gray matter thickness abnormalities as Cohen’s *d*, are mapped for nine different disorders, for which worldwide data were analyzed with the same harmonized methods. Although the cohorts included in the studies differed, as did the scanning sites and age ranges studied, some common and distinct patterns are apparent. Cortical maps for major depressive disorder (MDD), bipolar disorder (BD) and schizophrenia show gradually more extensive profiles of deficits. Across all disorders, the less prevalent disorders tend to show greater effects in the brain: the relatively subtle pattern of hippocampal-limbic deficits in MDD broadens to include frontal deficits in bipolar disorder (consistent with frontal lobe dysfunction and impaired self-control). In schizophrenia, deficits widen to include almost the entire cortex—only the primary visual cortex (specifically the calcarine cortex) failed to show thickness alterations in patients, after meta-analysis. Autism spectrum disorder (ASD) and the 22q deletion syndrome (22q11DS)—a risk condition for ASD—are associated with hypertrophy in frontal brain regions, while patients with obsessive-compulsive disorder (OCD) and alcohol use disorder tend to show deficits in frontal brain regions involved in self-control and inhibition. More refined analyses are now relating symptom domains to these and other brain metrics, within and across these and other disorders.

**Fig. 5:**
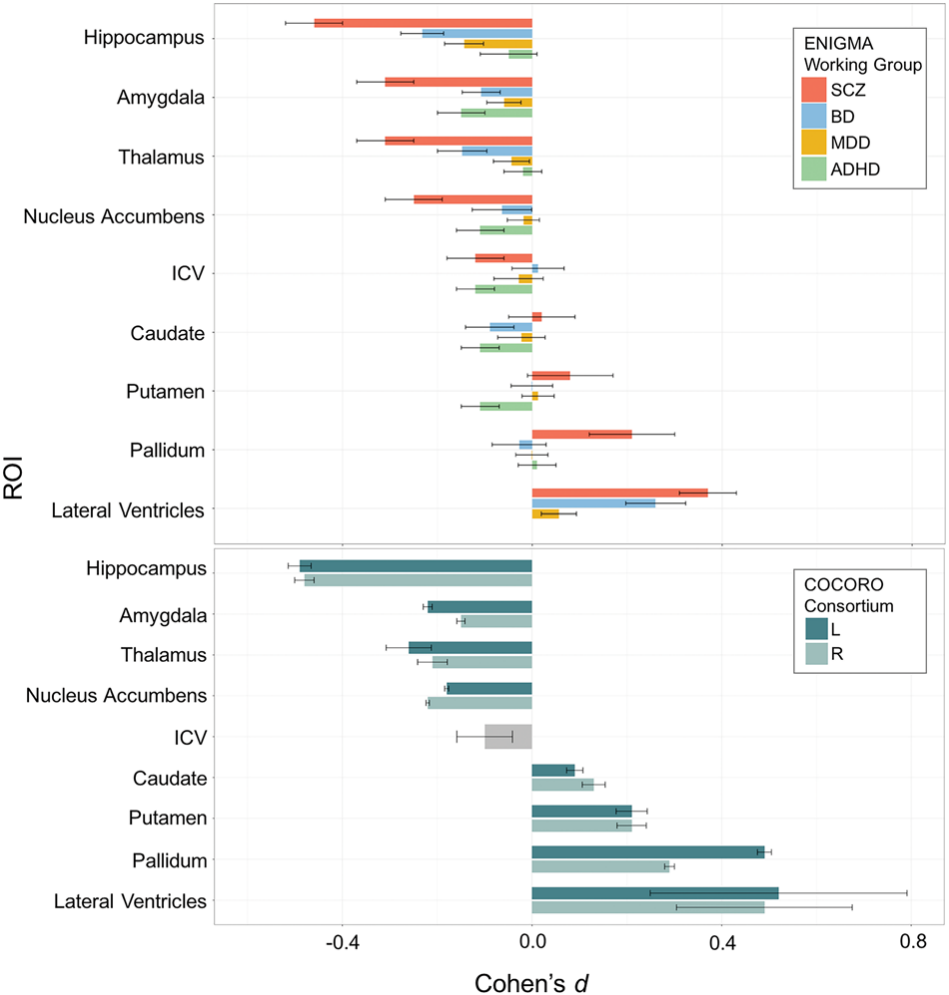
Subcortical abnormalities in schizophrenia, bipolar disorder, major depressive disorder, and ADHD. **a** ENIGMA’s publications of the three largest neuroimaging papers on schizophrenia (SCZ), bipolar disorder (BD), and major depressive disorder (MDD), suggested widespread cross-disorder differences in effects (van Erp et al.^54^, Hibar et al.^68^). By processing 21,199 people’s brain MRI scans consistently, we found greater brain structural abnormalities in SCZ and BD versus MDD, and a very different pattern in attention-deficit/hyperactivity disorder (ADHD; Hoogman et al.^7^). Subcortically, all three disorders involve hippocampal volume deficits—greatest in SCZ, least in MDD, and intermediate in BD. As a slightly simplified ‘rule of thumb’, the hippocampus, ventricles, thalamus, amygdala and nucleus accumbens show volume reductions in MDD that are around half the magnitude of those seen in BD, which in turn are about half the magnitude of those seen in SCZ. The basal ganglia are an exception to this rule—perhaps because some antipsychotic treatments have hypertrophic effects on the basal ganglia, leading to volume excesses in medicated patients. In ADHD, however, the amygdala, caudate and putamen, and nucleus accumbens all show deficits, as does ICV (ventricular data is not included here for ADHD, as it was not measured in the ADHD study). A web portal, the ENIGMA Viewer, provides access to these summary statistics from ENIGMA’s published studies of psychiatric and neurological disorders (http://enigma-viewer.org/About_the_projects.html). **b** Independent work by the Japanese Consortium, COCORO, found a very similar set of effect sizes for group differences in subcortical volumes between schizophrenia patients and matched controls.

Figures 4 and 5 present these cortical and subcortical findings alongside data from several other disorders. It is notable that these findings from ENIGMA^13,54^ were replicated in a large independent study by the Japanese COCORO Consortium^58^, and a recent Norwegian study of 16 cohorts by Alnæs et al.^59^. The convergence of all three studies, reviewed in Kochunov et al.^60^, represents a new level of rigor and reproducibility in a field where the existence of morphometric correlates of schizophrenia was once hotly debated^61^.

Brain alterations were also discovered in relation to clinical features of the disease. In follow-up analyses, Walton et al. found that positive symptom severity was negatively related to the thickness of the superior temporal gyrus bilaterally^62^, while the severity of negative symptoms was negatively related to the cortical thickness of several prefrontal regions and particularly the left medial orbitofrontal cortex (MOFC)^63^.

At this point it is worth considering the added value of other data modalities, such as diffusion MRI, which offers complementary information on microstructural abnormalities, especially in the WM, that are not detectable on standard anatomical MRI. ENIGMA’s Diffusion MRI working group, launched in 2012 with protocols for diffusion tensor imaging (DTI), published a series of papers on the heritability and reproducibility of DTI measures derived with a protocol based on tract-based spatial statistics^64–66^. Over ten of ENIGMA’s working groups have since used this protocol to rank effect sizes for DTI metrics across key WM tracts.

**Fig. 6:**
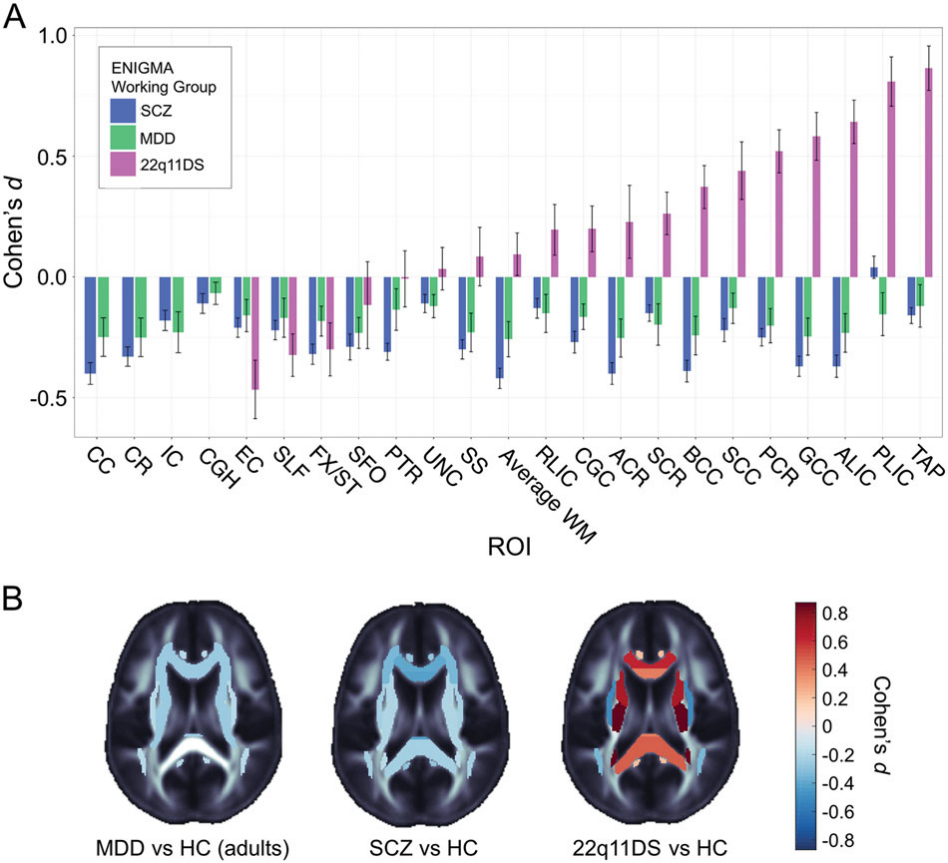
White matter microstructure in schizophrenia, major depressive disorder, and 22q11.2 deletion syndrome. **a** White matter microstructural abnormalities are shown, by tract, based on the largest-ever diffusion MRI studies of these three disorders. In schizophrenia (SCZ), fractional anisotropy, a measure of white matter microstructure, is lower in almost all individual regions, and in the full skeleton. In major depressive disorder (MDD), a weak pattern of effects is observed, again with MDD patients showing on average lower FA across the full white matter skeleton, when compared to controls. In comparisons between 22q11.2 deletion syndrome (22q11DS) and matched controls, by contrast, the average FA along the full white matter skeleton does not show systematic differences; instead, while some regions do show on average lower FA in affected individuals compared with controls, several white matter regions show higher FA. **b** Relative to appropriately matched groups of healthy controls (HC), group differences in fractional anisotropy are shown for ENIGMA’s studies of SCZ, MDD (both in adults), and 22q11.2 deletion syndrome. [Data adapted, with permission of the authors and publishers, from Kelly et al.^56^, van Velzen et al.^67^, and Villalón-Reina et al.^17^; a key to the tract names appears in the original papers; some tracts (i.e. the hippocampal portion of the cingulum) were omitted from the 22q11DS analysis as they were not consistently in the field of view for some cohorts of the working group].

Kelly et al. reported on widespread WM abnormalities in schizophrenia, pooling data from 2359 healthy controls and 1963 patients with SCZ from 29 independent international studies^56^. Significant reductions in fractional anisotropy (FA) in patients with SCZ were widespread across major WM fasciculi. While effect sizes varied by tract and included significant reductions in the anterior *corona radiata* (*d* = 0.40) and *corpus callosum* (*d* = 0.39, specifically its body (*d* = 0.39) and genu (*d* = 0.37)), effects were observed throughout the brain, with peak reductions observed for the entire WM skeleton (*d* = 0.42). Figure 6 shows these findings alongside data from two other disorders for which ENIGMA published large-scale DTI analyses, MDD^67^, and 22q11DS^17^.

#### ENIGMA-BD

Formed shortly after the Schizophrenia WG, and following similar protocols, the ENIGMA’s BD WG reported on cortical thickness and surface area measures using anatomical MRI data from 1837 adults with BD and 2582 healthy controls, from 28 international groups^68^. BD was associated with reduced cortical thickness in bilateral frontal, temporal and parietal regions, and particularly in the left pars opercularis (*d* = −0.29), the left fusiform gyrus (*d* = −0.29), and left rostral middle frontal cortex (*d* = −0.28). Interestingly, lithium use was associated with thicker cortex in several areas. The WG also examined case–control differences in subcortical volumes in 1710 patients with BD and 2594 healthy controls; they found that BD was associated with reductions in the volume of the hippocampus (*d* = −0.23) and the thalamus (*d* = −0.15), and with enlarged lateral ventricular volume (*d* = 0.26). A follow-up study, showed that when applied to regional cortical thickness, surface area, and subcortical volumes, machine learning methods (based on support vector machines) differentiated BD participants from controls with above chance accuracy even in a large and heterogeneous sample of 3020 participants from 13 ENIGMA cohorts worldwide^23^. Aggregate analyses of individual subject data yielded better performance than meta-analysis of site-level results. Age and exposure to anticonvulsants were associated with greater odds of correct classification. Although short of the 80% clinically relevant threshold, the 65.2% accuracy (0.71 ROC-AUC) is promising, as the study focused on a difficult to diagnose, highly heterogeneous condition and used only engineered features, not raw brain imaging data. ENIGMA’s multi-site design may also offer a more realistic assessment of “real-world” accuracy, by repeatedly leaving out different sites’ data for cross-validation. Future multisite brain-imaging machine learning studies will begin to move towards sharing of more detailed individual subject data, not only a selection of discrete features or site-level results derived from a single modality; unsupervised machine learning techniques may offer potential to better understand the heterogeneity in the disorder. The ENIGMA-BD DTI WG conducted both a mega- and meta-analysis of 3033 subjects (1482 BD and 1551 controls)^69^. Both analyses found lower FA in patients with BD compared with healthy controls in most brain regions, with the highest effect sizes in the corpus callosum and cingulum.

#### ENIGMA-MDD

Brain morphometric analyses conducted by the ENIGMA-MDD WG were based on MRI data from 1728 patients with MDD and 7199 controls for subcortical volumes^70^ and from 2148 patients with MDD and 7957 controls for cortical measures^3^. These studies found that patients with MDD had lower hippocampal volumes (*d* = −0.14), an effect driven by patients with recurrent illness (*d* = −0.17) and by patients with an adolescent (≤21 years) age of onset (*d* = −0.20). First-episode patients showed no subcortical volume differences compared to controls. Adult patients (>21 years) had reduced cortical thickness in bilateral orbitofrontal cortex (OFC), anterior and posterior cingulate cortex, insula, and temporal lobe regions (*d*’s: −0.10 to −0.14). In contrast, adolescent patients showed no differences in cortical thickness but showed lower total surface area, which seemed to be especially driven by lower surface area in frontal (medial OFC and superior frontal gyrus), visual, somatosensory, and motor areas (*d* = −0.26 to −0.57). Moreover, these differences in gray matter morphometry observed in MDD do not involve abnormal asymmetry, as shown in a joint study by the Laterality and the MDD WGs involving 2540 MDD individuals and 4230 controls, from 32 datasets^71^.

A follow-up analysis on a subset of these aforementioned data found that the brain MRIs of adult patients with MDD (18–75 years old) appeared, on average, 1.08 years older than those of controls (*d* = 0.14)^72^. This ‘brain age’ estimate was based on a machine learning algorithm trained to predict chronological age from morphometric data from 2188 controls across 19 cohorts and subsequently applied to hold-out data from 2126 healthy controls and 2675 people with MDD. The largest brain aging effects were observed in antidepressant users (+1.4 years; *d* = 0.15), currently depressed (+1.5 years; *d* = 0.18), and remitted patients (+2.2 years; *d* = 0.18), compared to controls. Within ENIGMA-MDD, Opel et al. also studied the effects of obesity on structural brain metrics of patients and controls (*N* = 6420)^73^. Obesity effects were not different between patients and controls, but there was a significant obesity by age interaction in relation to cortical thickness, with thinner cortices in older obese individuals. Cortical thickness deficits related to obesity were strongest in the temporal and frontal cortical regions, and overlapped with patterns observed in several neuropsychiatric disorders, but exceeded those found in MDD without regard for BMI—in terms of the effect sizes and range of structures affected. The magnitude of these effects suggests a need to better understand the connections between BMI, brain aging and mental health.

Capitalizing on the statistical power of ENIGMA to examine the role of risk factors, Frodl^74^ and Tozzi^75^ examined the association between retrospectively assessed childhood maltreatment (including emotional, physical and sexual abuse, or emotional and physical neglect), and brain morphometry in 3036 and 3872 individuals (aged 13–89) with and without MDD, respectively. Greater exposure to childhood maltreatment was associated with lower cortical thickness of the banks of the superior temporal sulcus and supramarginal gyrus, and with lower surface area across the whole brain and in the middle temporal gyrus. Sex differences were also observed: in females, greater maltreatment severity was associated with overall lower gray matter thickness and smaller caudate volumes, whereas in males, greater maltreatment severity was associated with lower thickness of the rostral anterior cingulate cortex.

In addition to these investigations of gray matter in MDD, a large-scale analysis of WM microstructure with DTI has also been completed, comparing 1305 adults and adolescents with MDD to 1602 healthy controls from 20 samples worldwide^67^. In adults with MDD, widespread lower FA values were found in 16 out of 25 WM tracts of interest (*d*’s = 0.12–0.26), with the largest differences in the corpus callosum and *corona radiata*. Widespread increased radial diffusivity (RD) was also observed (*d*’s = 0.12–0.18) and was driven by patients with recurrent MDD and an adult-onset of depression.

#### ENIGMA-PGC Post-Traumatic Stress Disorder

In partnership with the PGC, ENIGMA launched a WG on PTSD that has analyzed neuroimaging and clinical data from 1868 individuals (including 794 patients with PTSD) from 16 cohorts. In this first ENIGMA-PTSD study, Logue and colleagues found that patients with current PTSD had smaller hippocampal volumes (*d* = −0.17) compared to trauma-exposed controls^4^. Childhood trauma predicted smaller hippocampal volume (*d* = −0.17) independent of diagnosis. In a subsequent study, the WG found that cortical thickness in 3378 individuals (including 1309 patients with PTSD) was lower in PTSD in the orbitofrontal cortex, cingulate cortex, precuneus, insula, and lateral parietal cortices. In addition, a DTI meta-analysis of 3057 individuals (including 1405 patients with PTSD) from 25 cohorts found alterations in WM organization in the tapetum, a structure that connects the left and right hippocampus^76^. Structural covariance network analysis applied to data from 3505 individuals (including 1344 patients with PTSD), which examined correlated patterns of cortical thickness and surface area, found that PTSD is associated with network centrality features of the insula and visual association areas^77^. To extend these findings, ongoing studies are assessing cortical structure^78,79^ and hippocampal subfields in PTSD and MDD^80–83^, to better understand the pattern and regional specificity of hippocampal deficits in the two disorders, and whether these patterns coincide.

#### ENIGMA-Addictions/SUD

The ENIGMA-Addictions/SUDs WG has 33 participating sites, contributing MRI data from 12,347 individuals of whom 2277 are adult patients with SUD relating to one of five substances (alcohol, nicotine, cocaine, methamphetamine, or cannabis)^5,84,85^. In these data, Mackey^5^ observed lower cortical thickness/subcortical volume in cases relative to controls in regions that play key roles in evaluating reward (MOFC, amygdala), task monitoring (superior frontal cortex), attention (superior parietal cortex, posterior cingulate) and perception/regulation of internal body states (insula). While the most pervasive case–control differences appeared to be related to alcohol dependence, some effects were observed for substance dependence generally (e.g., the insula and MOFC). A support vector machine trained on cortical thickness and subcortical volume successfully classified set-aside test sets for both alcohol (ROC-AUC: 0.74–0.78; *p* < 0.0001) and nicotine dependence (ROC-AUC: 0.60–0.64; *p* < 0.0001), relative to non-dependent controls^5^. A separate meta-analysis also compared the effect size of addiction-related brain impairment to that of other psychiatric disorders: effect sizes of alcohol-related brain differences in subcortical brain regions were equivalent to those reported for schizophrenia^86^.

#### ENIGMA-Obsessive-Compulsive Disorder

The ENIGMA’s OCD WG grew out of a previously established consortium (the OCD Brain Imaging Consortium, or OBIC)^87^, and has published the largest studies to date of brain structure in adult and pediatric OCD, using both meta- and mega-analytic approaches^6,88^. The first study analyzed MRI scans from 1830 patients diagnosed with OCD and 1759 controls across 35 cohorts from 26 sites worldwide^88^. Unmedicated pediatric OCD patients demonstrated larger thalamic volumes, while the pallidum was enlarged in adult OCD patients with disease onset at childhood. Adult OCD patients also had significantly smaller hippocampal volumes (*d* = −0.13), with stronger effects in medicated patients with adult-onset OCD compared to healthy controls (*d* = −0.29). A cortical study included data from 1905 patients diagnosed with OCD and 1760 healthy controls across 38 cohorts from 27 sites worldwide. In adult patients diagnosed with OCD versus controls, significantly smaller surface area of the transverse temporal cortex (*d* = −0.16) and a thinner inferior parietal cortex (*d* = −0.14) were found. Medicated adult patients with OCD also showed thinner cortices throughout the brain (Cohen’s *d* effect sizes varied between −0.10 and −0.26). Pediatric patients with OCD showed significantly thinner inferior and superior parietal cortices (*d*’s = −0.24 to −0.31), but none of the regions analyzed showed significant differences in cortical surface area. However, *medicated* pediatric patients with OCD had smaller surface area in frontal regions (*d*’s = −0.27 to −0.33), that may indicate a delayed cortical maturation. The absence of cortical surface area abnormalities in adult patients with a childhood onset of OCD could indicate a normalization of these abnormalities—a hypothesis that is now being explored with longitudinal data collection.

To assess whether the anatomical differences could be used to create a neuroimaging biomarker for OCD, a machine learning analysis of the cortical and subcortical data was performed with 2304 OCD patients and 2068 controls. Classification performance across ten different machine and deep learning approaches was poor. With site-stratified cross-validation, the ROC-AUC ranged between 0.57 and 0.62. The performance dropped to chance level when leave-one-site-out cross-validation was used, with classification performance between 0.51 and 0.54. This indicates that these anatomical brain features do not provide a biomarker for OCD. But when patients were stratified according to whether they had used medication, classification performance improved remarkably. Medicated OCD patients and controls could then be distinguished with 0.73, unmedicated OCD and controls with 0.61, and medicated and unmedicated OCD patients with 0.86 ROC-AUC. These multivariate results therefore mirror the univariate results, and highlight that medication use is associated with large differences in brain anatomy^89^.

The OCD WG, in conjunction with the Laterality WG, studied brain asymmetry in OCD using 16 pediatric datasets (501 patients with OCD and 439 healthy controls), and 30 adult datasets (1777 patients and 1654 controls)^90^. In the pediatric datasets, the largest case–control differences were observed for volume asymmetry of the thalamus (more leftward in patients compared to controls; *d* = 0.19) and the pallidum (less leftward in patients compared to controls; *d* = −0.21). No asymmetry differences were found in the adult datasets. These findings may reflect altered neurodevelopmental processes in OCD, affecting cortico-striato-thalamo-cortical circuitry, which is involved in a wide range of cognitive, motivational and emotional processes.

#### ENIGMA-Attention-Deficit/Hyperactivity Disorder

ENIGMA’s ADHD WG has analyzed data from up to 2264 participants with ADHD and 1934 controls from up to 36 sites (age range: 4–63 years; 66% males)^91^. Volumes of the nucleus accumbens (*d* = −0.15), amygdala (*d* = −0.19), caudate (*d* = −0.11), hippocampus (*d* = −0.11), putamen (*d* = −0.14), and ICV (*d* = −0.10) were smaller in cases relative to controls. Effect sizes were highest in children. No statistically significant univariate case/control differences were detected in adults. Volume differences were found to have similar effect sizes in those treated with psychostimulant medication and those naïve to psychostimulants. Bioinformatics analyses suggested that the selective subcortical brain region vulnerability was associated with differential expression of oxidative stress, neurodevelopment and autophagy pathways^92^.

The ENIGMA-ADHD WG was the first WG in ENIGMA to perform a detailed investigation of the case-control effects on the cerebellum. Differential age trajectories were identified for children with ADHD when compared with typically developing children for the *corpus medullare*^93^.

In an analysis of the cerebral cortex, lower surface area values were found, on average, in children with ADHD, mainly in frontal, cingulate, and temporal regions; the largest effect was for total surface area (*d* = −0.21). Fusiform gyrus and temporal pole cortical thickness were also lower in children with ADHD. All effects were most pronounced in early childhood. Neither surface area nor thickness differences were found in the adolescent or adult groups^7^, but machine learning analyses supported the hypothesis that the case–control differences observed in childhood could be detected in adulthood^94^. Importantly, many of the same surface area features were associated with subclinical ADHD symptoms in children from the general population that do not have a clinical psychiatric diagnosis. Several of the observed brain alterations fulfilled many of the criteria of ‘endophenotypes’ (An endophenotype is a trait, such as brain structure or function, related to the biological process of a disorder; to qualify as an endophenotype, the trait, should be heritable, co-segregate with an illness, yet be present even when the disease is not, and be found in non-affected family members at a higher rate than in the general population^95,96^), as they were also seen in unaffected siblings of people with ADHD in a subsample analysis of the cortical features. The stronger effects in children may reflect a developmental delay, perhaps due in part to genetic risk factors, given recent findings of overlap between the genetic contributions to ADHD and to subcortical volumes^13,47^.

### ENIGMA-Autism Spectrum Disorders

The ENIGMA-ASD WG published the largest neuroimaging study of autism analyzing data from 1571 participants with ASD and 1651 controls, from 49 sites worldwide (ages 2–64 years)^8^. Unlike most of the disorders discussed so far, the direction of effects seen in ASD varied by brain region, and did so across the age span analyzed. ASD was associated with larger lateral ventricle and intracranial volumes, greater frontal cortical thickness and lower temporal cortical thickness (*d* = −0.21 to 0.20). Participants with ASD also had, on average, lower subcortical volumes for the pallidum, putamen, amygdala, and nucleus accumbens. Post hoc fractional polynomial analyses showed a sharp increase in volumes in the same regions in childhood, peaking in adolescence and decreasing again in adulthood. Overall, patients with ASD showed altered morphometry in the cognitive and affective associated-regions of the striatum, frontal cortex, and temporal cortex.

The ASD group worked together with the Laterality group to produce the largest ever study of brain asymmetry in ASD, involving 1774 patients and 1809 controls, from 54 datasets^97^. Generally, subtle but widespread reductions of cortical thickness asymmetries were present in patients with ASD compared to controls, as well as volume asymmetry of the putamen, and surface area asymmetry of the MOFC (the strongest effect had Cohen’s *d* = −0.16). Altered lateralized neurodevelopment may, therefore, be a feature of ASD, affecting widespread cortical regions with diverse functions.

### Neurogenetic disorders, CNV, and rare neurodevelopmental conditions

Several neurodevelopmental disorders arise due to the abnormal duplication or deletion of segments of the genome. ENIGMA has dedicated WGs studying 22q11DS, Gaucher’s disease, and Hepatic Glycogen storage disease^98,99^, along with a CNV WG meta-analyzing imaging data from carriers of several other CNVs^53,100^. Here, we focus on the work of the two most established groups, that examine carriers of 22q11.2 deletions and other CNVs.

#### ENIGMA-22q11.2 Deletion Syndrome

22q11DS is associated with a 20-fold increased risk for psychosis, and an elevated risk for developmental neuropsychiatric disorders such as ASD. 22q11DS provides a ‘genetics-first’ framework to study the brain markers underlying complex psychiatric phenotypes. The ENIGMA-22q11DS working group analyzed the largest dataset to date of brain images from patients with 22q11DS from 10 cohorts including 466 individuals with 22q11DS and 374 matched controls. Compared to controls, 22q11DS individuals showed overall thicker cortical gray matter (left/right hemispheres: Cohen’s *d* = 0.61/0.65), but pervasive reductions in cortical area (left/right hemispheres: *d* = −1.01/−1.02), with specific anatomic patterns. Machine learning methods were applied to the cortical thickness and area measures to achieve a high accuracy (sensitivity 94.2%; specificity 93.3%) in classifying 22q11DS cases and controls^10^. ENIGMA subcortical shape analysis pipelines also identified complex structural differences across many subcortical structures between individuals with 22q11DS and controls^101^. Analysis of diffusion MRI from the same subjects (*N* = 594) revealed abnormalities in the corpus callosum, superior longitudinal fasciculus, and *corona radiata*^17^. Ongoing work uses more advanced imaging protocols^17^—including ‘multishell’ diffusion protocols that allow for the estimation of biophysical compartments in the tissue—to test hypotheses about specific cellular processes and specific fiber tracts that may be especially vulnerable in 22q11DS (e.g., the corpus callosum), as well as fiber tracts that appear to be relatively spared (e.g., the cortico-fugal tracts^17^).

#### ENIGMA-Copy Number Variations

This WG was set up to examine the effect of rare CNVs, risk factors for a variety of neuropsychiatric disorders, on brain structure. Due to their low prevalence^102,103^, their effects on the brain have been hard to establish. Sønderby and colleagues focused on the 16p11.2 distal CNV that predisposes to psychiatric conditions including ASD and schizophrenia. ENIGMA (including the 16p11.2 European Consortium) and deCODE datasets were combined to compare subcortical brain volumes of carriers of 15 16p11.2 distal deletion and 18 duplication to 7714 non-carriers which led to the discovery of negative dose-response associations with copy number on intracranial volume and regional accumbens, caudate, pallidum and putamen volumes—suggesting a neuropathological pattern that may underlie the neurodevelopmental syndromes^53^. A further study^100^ including the UK Biobank assessed the association of the 15q11.2 CNV with cognition and cortical and subcortical morphology in more than 45,000 individuals from 38 datasets (203 individuals with a 15q11.2 deletion, 45,247 non-carriers, and 306 duplication carriers). The authors found a clear pattern of widespread poorer cognitive performance, smaller surface area and thicker cortices for deletion carriers compared to non-carriers and duplication carriers, particularly across the frontal lobe, anterior cingulate and pre/postcentral gyri. The pattern of results fits well with known molecular functions of the genes in the 15q11 region and suggests involvement of these genes in neuronal plasticity and cortical development. Thus, the results from ENIGMA-CNV have shown that several CNVs cause abnormal brain patterns and inform on genetically determined variation in brain development and their relation to neurodevelopmental disorders. Additional studies on other CNVs are in progress.

### Newly established working groups

In the last two years, seven additional ENIGMA WGs have formed to study specific disorders and important transdiagnostic conditions: anxiety disorders, suicidal thoughts and behavior, sleep and insomnia, eating disorders (including bulimia and anorexia nervosa subgroups^104^), irritability, antisocial behavior, and dissociative identity disorder. The starting point of the anxiety group was an international voxel-based morphometry mega-analysis on social anxiety disorder^105^, supported by findings demonstrating that structural brain alterations related to social anxiety run in families^106^. At present, the anxiety WG has four subgroups including over 5000 patients: besides social anxiety disorder (1250 patients)^107^, there are groups devoted to generalized anxiety disorder (1329 patients), panic disorder (1300 patients), and specific phobia (1224 patients), allowing for disorder-specific and cross-disorder comparisons. The antisocial behavior WG aims to clarify how conduct disorder, psychopathy, and antisocial personality disorder relate to differences in brain structure, function, and connectivity. Its goals include examination of different phenotypes (e.g., reactive vs proactive aggression), population-based samples with dimensional measures of antisocial behavior, and genetic data from case–control and population-based studies.

Building on the promising findings from the psychiatric WGs, ENIGMA established seven WGs studying specific conditions in neurology and cancer-related cognitive impairment: epilepsy, traumatic brain injury, Parkinson’s disease, neuro-HIV, ataxia, stroke recovery, and cancer/chemotherapy effects on the brain^108,109^.

#### ENIGMA-Epilepsy

The ENIGMA-Epilepsy WG combined data from 24 centers across 14 countries to create the largest neuroimaging study to date of epilepsy^9^. Data from 2149 individuals with epilepsy were divided into four common epilepsy syndromes: idiopathic generalized epilepsies (*N* = 367), mesial temporal lobe epilepsies with hippocampal sclerosis (MTLE; left, *N* = 415; right, *N* = 339), and all other epilepsies in aggregate (*N* = 1026), compared to 1727 matched healthy controls. Compared to controls, all epilepsy groups showed lower volume in the right thalamus (*d* = −0.24 to −0.73), and lower thickness in the precentral gyri bilaterally (*d* = −0.34 to −0.52). Both MTLE subgroups also showed profound volume reduction in the ipsilateral hippocampus (*d* = −1.73 to −1.91), and lower thickness in cortical regions, including the precentral and paracentral gyri (*d* = −0.36 to −0.52) compared to controls. Notably, the effect sizes for cortical differences in this neurological disorder were much greater than those seen in all complex psychiatric disorders. In an approach known as ‘virtual histology’, a follow-up study^110^ overlaid the cortical deficit maps on gene-expression data from the Allen Brain Atlas, and detected enrichment for microglial markers in regions with greater deficits. The WG is currently combining DTI data and exploring putative neuroanatomical biomarkers of medication treatment resistance and post-operative outcomes.

#### ENIGMA-Brain Injury

ENIGMA’s Brain Injury WG^111^ combines data from 72 centers, and is organized into ten separate subgroups that focus on (1) acute mild traumatic brain injury (TBI), (2) adult moderate/severe TBI^112^, (3) pediatric moderate/severe TBI^113,114^, (4) military-related brain injury^115–118^, (5) sports-related concussion^119^, (6) intimate partner violence^120^, (7) MR spectroscopy^121^, (8) arterial spin labeling, (9) resting state fMRI, and (10) cognitive endpoints. These groups have recently started-up compared to other ENIGMA WGs, but are rapidly expanding in membership and focus. In addition to meta- and mega-analyses of relevant existing datasets, the Brain Injury WGs endeavor to further extend efforts to promote increased consistency in prospective data collection, both in terms of imaging data and associated cognitive outcome data. Additionally, the WGs are engaged in the development of novel pipelines and analytic tools that address brain-injury specific issues or incorporate sequences or techniques that are potentially useful in addressing injury associated pathology. For example, future planned studies will compute structural pathology profiles for individual TBI patients, including (i) mapping of the heterogeneous lesions using advanced lesion mapping methods (such as disconnectome symptom mapping), (ii) accurate quantification of brain atrophy (of the different brain regions) using tensor based morphometry, and (iii) identification of subject-specific epicenters best predictive of neurodegeneration using network spread models. Finally, the Brain Injury WGs will interface with other disease-specific WGs where comorbidity with brain injury is high (e.g., substance use, PTSD, MDD, ADHD), as well as with methods-focused WGs (e.g., diffusion imaging, etc.). A preliminary report on 117 participants with military-relevant blast-related versus 227 participants with non-blast related injury revealed higher FA in veterans and service members with blast-related injuries, and altered subcortical volumes in the group with military TBI overall^117^. Work is ongoing to study the effects of injuries sustained during and outside deployment, and severity and mechanisms of injury.

#### ENIGMA-Parkinson’s Disease

ENIGMA’s Parkinson’s disease WG has analyzed scans from 11 cohorts spanning 10 countries including 1288 patients with PD and 679 controls (age: 20–89 years)^122,123^. A PD diagnosis was associated with moderately larger thalamic volumes (left: *d* = 0.29; right: *d* = 0.17) and smaller pallidal volumes (left: *d* = −0.25; right: *d* = −0.21). There was also widespread and lower cortical thickness in PD patients, while sparing the limbic and insular cortices. Ongoing work on a larger sample is relating brain structure and WM microstructure to disease severity, medication status and history and duration of the illness as modifiers of these robust differences between patients and controls.

#### ENIGMA-Human Immunodeficiency Virus

The availability of combination antiretroviral therapy (cART) has now transformed HIV-infection from a possibly fatal diagnosis to a chronic condition, allowing for viral suppression and stable immune function; however, despite inconsistencies in neuroimaging studies, neurological symptoms and consequences persist. This WG has pooled data from 12 independent neuro-HIV studies from Africa, Asia, Australia, Europe, and North America; volume estimates for eight subcortical brain regions were extracted from anatomical MRI from 1044 HIV + adults (age: 22–81 years) to identify associations with plasma markers reflecting immunosuppression (CD4+ T-cell count) or viral load^124^. Across participants, lower current CD4+ count was associated with smaller hippocampal and thalamic volumes. A detectable viral load was also associated with smaller hippocampal (*d* = 0.24) and amygdalar volumes (*d* = 0.18), supporting the importance of achieving viral suppression and immune restoration. These limbic effects are in contrast to many of the early neuro-HIV findings that focused on basal ganglia structures, yet we found the limbic associations were largely driven by participants on cART, while basal ganglia effects (putamen) were detected in the subset of participants not on cART. These findings demonstrate the continuing effects of HIV on the brain in the current “cART era”. Alterations in brain structures that are essential for learning and memory has clinical significance given mounting evidence of HIV-associated deficits in these cognitive domains among older HIV+ adults, and the possibility that HIV may contribute to abnormal brain aging^125^.

#### ENIGMA-Ataxia

This WG includes 21 sites pooling data from more than 750 individuals with inherited ataxias, including Friedreich Ataxia and Spinocerebellar Ataxia (SCA) 1, 2, 3, 6, and 7 (the poly-glutamine SCAs), alongside over 800 controls. This group is undertaking optimization and standardization of protocols for cerebellar voxel-based morphometry and parcellation, upper spinal cord cross-sectional area, and brainstem volume, in line with the key regions of pathology in these diseases. Preliminary work indicates that gray matter degeneration principally impacts the cerebellar anterior lobe in Friedreich ataxia, while all areas of the cerebellum are affected in the poly-glutamine SCAs. However, both the magnitude and pattern of cerebellar gray matter degeneration are distinct across these diseases and evolve with disease progression and severity.

#### ENIGMA-Stroke Recovery

The ENIGMA-Stroke recovery WG has addressed a major gap in stroke research relating to the large-scale definition of lesion masks. Researchers in this WG have released a public archive of 304 T1-weighted MRIs with manually segmented stroke lesion masks^126^ (https://www.icpsr.umich.edu/icpsrweb/ADDEP/studies/36684), and developed open-source software^127^ and analyses specific for scalable^128^, reproducible lesion analyses (https://github.com/npnl/PALS). In addition to this major methodological contribution they have analyzed data from 629 participants from 22 sites worldwide to identify reliable predictors of motor function after stroke^129,130^. They found that motor-related subcortical volumes in the basal ganglia and thalamus are positively associated with post-stroke motor performance, and depend on impairment severity, time since stroke, and lesion laterality. In contrast, enlarged lateral ventricles are associated with worse post-stroke motor outcomes. The group now has data from 1625 participants from 32 sites worldwide, and ongoing work in the group focuses on quantifying lesion overlap with major motor-related structures, such as the corticospinal tracts and subcortical regions^131,132^, and relating these measures with subcortical volumetric measures to motor outcomes^133^.

### ENIGMA-methods focused working groups

The ENIGMA Consortium functions as a driving force for the development, validation and implementation of novel methods to address the complexities of analyses of large imaging datasets and to derive more mechanistic insights into the processes that underpin variation in brain organization in health and disease. To achieve this, ENIGMA has dedicated WGs focused on the development of more innovative pipelines for data analyses to be applied for various dataset worldwide. The ENIGMA Diffusion MRI WG on DTI is one of the most long-standing. DTI offers information on microstructural abnormalities that are not detectable on standard anatomical MRI. As mentioned earlier, this WG has published a series of papers on the heritability and reproducibility of DTI measures derived with a custom protocol based on tract-based spatial statistics^64,134^. Diagnosis-based WGs have used this protocol to rank effect sizes for DTI metrics as previously described or are undertaking similar studies including in 22q11DS^17^, epilepsy, PTSD^76^, military TBI^113^, HIV^135^, and OCD^136^.

Other methodological WGs have focused on anatomical shape analyses that enable a more precise characterization of regional brain alterations thus resolving subregional effects in the basal ganglia, amygdala, and hippocampus^55,85,137–142^. Other approaches currently used in ENIGMA include brain structural covariance analysis graph theory approach for intra-individual brain structural covariance networks in OCD^77,143^, sulcal morphometry^144^, hippocampal subfield analysis^80–82,145^ and disease effects on lateralization (in OCD, MDD, and ASD)^71,90,97^. More recently, ENIGMA’s Brain Age WG was formed to apply various algorithmic estimators of ‘brain age’ across several ENIGMA WGs^72^. From the ENIGMA-Brain Injury group, the MR spectroscopy (MRS) WG has formed to focus on the harmonization of MRS data which could reach across other WGs in the future.

### The impact of ENIGMA

The ENIGMA Consortium has been a driving force in the field of neuroscience by making substantial contributions to the science of brain variation and shaping the working practices of the field at various levels. In reflecting on the key achievements, three areas stand out:

#### Promoting robustness and reproducibility

ENIGMA’s “big data” approach to neuroimaging addresses directly the reproducibility challenges that plague many areas of biomedical science—including neuroscience^146–148^. Neuroimaging has received considerable scrutiny regarding the reliability of published findings, given the literature replete with studies based on small samples and seemingly unlimited methodological freedom^149,150^. Many other approaches also aim to tackle this reproducibility crisis, by building data repositories that can be accessed for replication^151–153^; yet ENIGMA offers an opportunity to collaborate with teams of diverse experts irrespective of whether or not any data is shared. In one recent study by ENIGMA’s Laterality group, the authors examined brain asymmetry in 99 MRI datasets worldwide (from *N* = 17,141 people) and found that, as expected, the reproducibility of findings increased with the effect size and sample size, in a setting that was free from publication bias (data available at: http://conxz.net/neurohemi/)^18,154^. For example, for effect sizes of *d* ≥ 0.6, the reproducibility rate was higher than 90% even when including the datasets with sample sizes as low as 15, while it was impossible to obtain 70% reproducibility for small effects of *d* < 0.2, even with a relatively large minimum sample size threshold of 500. The unprecedented size of the datasets analyzed across ENIGMA boosts statistical power to detect the effects of disease and their moderators^73,155^. Through data sharing, investigators can now identify patterns of brain abnormalities that consistently characterize disorders or clinical syndromes, while assessing their reproducibility across continents. This is exemplified by the close match between the schizophrenia findings by ENIGMA^54,60^ and independent work by the Japanese Consortium, COCORO^58^ and a recent Norwegian study of 16 cohorts by Alnæs^59^. In all three studies, schizophrenia patients showed enlargement of the lateral ventricles, pallidum, putamen, and caudate, and volume reduction in the hippocampus, amygdala, thalamus and accumbens, with a strong agreement in the magnitude and rank order of effects from highest to least group difference. Similarly, a recent GWAS study of the UK Biobank dataset^156^ was able to replicate the majority of the genetic loci discovered by ENIGMA in two separate GWAS of subcortical volumes^24,25^. Thus, the international, multi-site nature of ENIGMA studies likely promotes representative findings that are widely generalizable. Meanwhile, the larger and more diverse samples are valuable resources for understanding the heterogeneity across different studies, and may provide new insights into the reproducibility issue faced by the neuroimaging community. Moreover, ENIGMA offers a platform for investigators to converge on methods for sharing and analyzing data acceptable to the community.

ENIGMA also offers new opportunities to change the landscape for how data can be used. In current research practices, a great resource of data remains largely untapped that is often known as “long-tail” data: data sets collected in individual laboratories that accumulate over many years and funding cycles^157^. Much valuable data remains dormant (and unpublished) due to a lack of personnel and time to analyze it, and this is going to increase with studies including larger samples than before.

Efforts through ENIGMA to leverage ‘dormant’ data in labs throughout the world have at least three important advantages. First, data sharing increases the scope of the science, enhancing opportunities for analyses not otherwise possible with small sample sizes. Second, data sharing naturally engages scientists from distinct disciplines—a crucial step for advancing the clinical neurosciences^158^. A final benefit that is sometimes overlooked in global scientific collaborations is their power to build and enhance diplomatic relations and transcend political conflicts between nations^159^. With representation from 43 countries—some of which have minimal diplomatic ties—collaborations are not only constructive in terms of collective problem solving, but they also build connections between high income countries and the poorest nations across the globe and to build capacity in the latter^159^.

#### Setting methodological standards

The ENIGMA Consortium has provided a blueprint for multi-site standardization in terms of mining legacy neuroimaging and genetic data. The success of this approach is obvious when considering the volume of over 50 published works that has relied on the ENIGMA pipelines. Furthermore, funding bodies, such as the National Institutes of Health in the United States, have gained interest in such approaches; program announcements requesting applications on aggregating existing biomedical data, or making use of existing resources, have become increasingly common. Moving forward, ENIGMA remains a test-bed of unprecedented scale and power for developing and benchmarking novel analytic methods. This contribution is of paramount importance as advanced statistical modeling and bioinformatics become essential for analytic pipelines. In efforts complementary to the technical advances made in multisite data collection initiatives such as the Alzheimer’s Disease Neuroimaging Initiative, the Human Connectome Project, and the UK Biobank, ENIGMA’s studies have required us to continually develop new scientific approaches to analyze data from diverse and independent populations. ENIGMA has made several technical advances that may be adapted to other domains, including creating, adapting, and extensively testing harmonized methods for distributed analysis, meta-analysis, and cross-site data integration (see Supplementary Appendix A: Technical Contributions of ENIGMA).

#### Driving discovery

Neuroimaging and genetics are fields of both large and small effects. For common, complex chronic diseases, effects on brain metrics can be very subtle, but for rare monogenic disorders and across the field of neurology—including epilepsy, brain injury, stroke and neuro-oncology, disease effects can be relatively large (although not exclusively). In the 10 years since ENIGMA was founded, the primary lesson has been on the power of worldwide collaboration to discern subtle patterns in brain data, and advance neuroscience beyond the capacity of any one group of researchers collecting data on their own. New discoveries regarding the factors that influence brain organization and its association with health and disease are predicated on having adequate statistical power and on developing new neuroimaging approaches aiming to lead to more mechanistic explanations of the multi-scale organization of the brain.

#### Challenges and future directions

Even given the advances made through ENIGMA during its first decade, as a growing consortium, ENIGMA faces important challenges. Thus far, ENIGMA has largely relied on existing data, which implies a degree of heterogeneity with respect to phenotyping—including clinical assessments, scanners and imaging protocols. Another limitation of this type of data is that the depth of phenotyping varies across centers, which can lead to a limited set of clinical and other scales shared by all centers. As we discuss below, ENIGMA is now beginning to address these limitations with a series of newly funded and planned studies^111,112,114,118–121^. The paucity of longitudinal data in the literature is also reflected within ENIGMA, which includes a limited number of longitudinal studies. Consequently, the data-driven approach used in ENIGMA is complementary, but not always superior, to well-designed, hypothesis-driven, smaller-scale prospective single-center or multi-center studies with in-depth phenotyping.

#### Extending imaging modalities and computational approaches

ENIGMA’s future developments will include the coordinated analyses of new data modalities (such as resting state and task-related functional MRI^160–163^, as well as geostatistical and mobile sensor data), and deeper or more refined analyses of current imaging modalities. Diffusion MRI, in particular, is moving towards multi-shell protocols that can better differentiate cellular and microstructural sources of variance that may explain patterns observed with DTI^17^. Multimodal projects that pool data across imaging modalities are likely to boost the accuracy of machine learning methods for differential diagnosis, outcome prediction, and subtyping. Unsupervised learning—applied to imaging and clinical data—may also help to identify homogeneous subgroups within and across disorders. Deep learning, for example, benefits from very large datasets, such as those analyzed in ENIGMA, and these and other artificial intelligence methods show promise in identifying unsuspected features and patterns in images beyond those derived using traditional methods. From its inception, ENIGMA has accommodated varying data sharing practices across institutions and countries, has used strategies (such as meta-analysis) to overcome some of these, and is working with field experts on novel strategies (like COINSTAC or other distributed analysis approaches)^164^ to allow for more powerful analysis without sending data around the globe. On the ‘omics’ side, whole genome sequencing promises to refine our understanding of causal loci across all phenotypes, from plasma markers and brain metrics to environmental exposure and clinical measures of disease burden.

#### Cross-disorder analyses

**Fig. 7:**
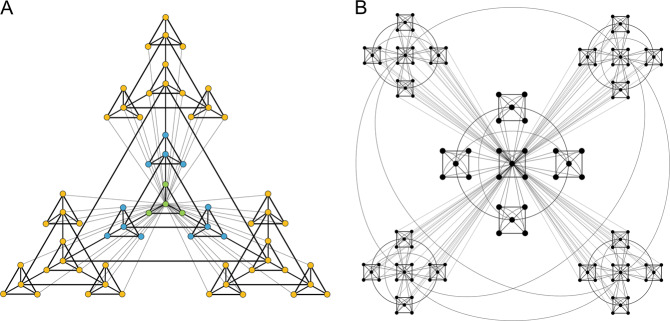
Topology of large-scale scientific collaboration. **a** The topology of scientific collaboration in ENIGMA has some properties that resemble a modular hierarchical network (Ravasz and Barabasi^170^, Slaughter^171^). In this diagram (**a**), nodes represent individual scientists working on a project, and links denote active scientific collaborations (that might result in co-authored publications, like this review, for example). ENIGMA’s WGs resemble the yellow sets of nodes: guided by a small group of WG chairs, several clusters of scientists coordinate projects applying various methods to the same datasets (e.g., MRI and DTI meta-analysis, machine learning, and modeling of clinical outcomes). WGs study different disorders with the same harmonized methods, enabling to cross-disorder collaborations across WGs. The modular organization allows independent and coordinated projects to proceed in parallel, distributing work and coordination, without requiring a central hub for all communication. Real clusters may differ in their number of members and links [(**b**) shows a different graph with a similar hierarchical modular form], and may change dynamically over time as new groups and projects form and projects end.

ENIGMA has recently created cross-disorder groups to answer transdiagnostic questions that draw on data from multiple WGs^165^. An exemplar of this approach is the newly formed ENIGMA-Relatives WG which examines brain organization in the unaffected first-degree relatives of patients with psychiatric disorders. The first study from this group focused on identifying common and distinct anatomical patterns in patients with SCZ (*N* = 1016) or BD (*N* = 666) and their unaffected relatives (*N* for SCZ relatives = 1228 and for BD relatives = 852)^11^. A remarkable finding from this study is that the first-degree relatives of BD patients had larger ICV compared to controls (*d* = 0.16) while first-degree relatives of SCZ patients had smaller ICV and lower cortical thickness, and when controlling for ICV, had regionally smaller thalamic volumes. Other newly formed groups aim to aggregate data across the spectrum of mental illness that may be prone to similar symptoms and outcomes, such as suicidal ideations and actions. Cross-disorder initiatives have formed within ENIGMA as partnerships between existing group members. The topology of collaboration (Fig. 7) includes working partnerships between group members working on similar problems, providing natural connections between topics of study of the different groups.

#### Sex differences

ENIGMA’s sex differences Initiative is probing disease WG datasets to better understand sex disparities in risk factors, disease effects, or outcomes and their relationship with brain organization. In a new initiative, the ENIGMA-Transgender WG is contributing additional insights with respect to the biological underpinnings of sex assigned at birth versus gender identity^166^. The first study from this group was based on more than 800 scans and pooled various MRI-based measures (cortical thickness, surface area, and volume) across eight international sites. While effects varied depending on the morphometric measure applied and the brain regions considered, a general pattern emerged: transgender men (assigned female at birth) mostly resemble cis-gender women, whereas transgender women (assigned male at birth) range between cis-gender men and cis-gender women^167^. Ongoing initiatives examining sex-differences focus on sex-specific GWAS studies, and developmental and aging trajectories.

#### Global health disparities

Health disparities, including those that exist in low and middle income countries, are also a topic of great interest for ENIGMA, as prevalence, treatments, and access to healthcare varies within and across countries. While the analyses in ENIGMA so far tend to show cross-national agreement in brain signatures and associated genetic loci of various psychiatric diseases, more in-depth phenotyping may reveal circumstances where risk factors apply more strongly to specific ethnic or sociodemographic groups, and means to remediate them, consistent with the concept of precision public health.

In closing, we reiterate ENIGMA’s mission statement, which reads: “Individually, we contribute little to the quest for truth, but working together, the whole vast world of science is within our reach.” (Aristotle, 350 BCE)^168^.

## Supplementary information

Supplementary Appendix
