# Supplementary material for: ENIGMA and global neuroscience: A decade of large-scale studies of the brain in health and disease across more than 40 countries
Source: Transl Psychiatry. 2020 Mar 20;10:100. doi: 10.1038/s41398-020-0705-1 (PMC7083923; doi:10.1038/s41398-020-0705-1)
Supplement: Supplementary file 1 — Supplementary Appendix [file 41398_2020_705_MOESM1_ESM.pdf]

Supplementary Appendix

ENIGMA and Global Neuroscience: A Decade of Large-Scale Studies of the Brain in Health and Disease across more than 40 Countries

Correspondence: Paul M. Thompson, pthomp@usc.edu

A. Technical Contributions of ENIGMA

Technical Contributions of ENIGMA. ENIGMA's ongoing technical contributions include large-scale testing and refinements of protocols to standardize the pre-processing, harmonization, and analysis of multiple types of imaging and genetic data, as well as new data modalities such as magnetic resonance spectroscopy (MRS; Bartnik-Olson 2019) and EEG (Smit 2018). These contributions have simplified the process for merging and comparing multi-source brain data in large-scale analyses across fields of psychiatry, neurology, and developmental neuroscience.

I. **Genetics.** ENIGMA's genetics protocols (http://enigma.ini.usc.edu/protocols/genetics-protocols/) enabled over 50 sites to participate, on an unprecedented scale, in large-scale GWAS studies of brain-derived MRI measures including subcortical and cortical volumes, ICV (Stein 2012; Hibar 2015; Satizabal 2019; Grasby 2019; Hofer 2019), as well as EEG (Smit 2018). The genetic analysis protocols include methods to impute data from multiple genotyping chips, to conduct statistical association tests across the genome (in both unrelated samples and family designs), and meta-analysis protocols for combining genome-wide association data from many sites. Extensive quality control procedures are included. A recent extension of this work includes protocols for genome-wide epigenetic analysis (EWAS) of brain traits, which were applied to discover loci where methylation levels relate to hippocampal volumes by pooling evidence across multiple datasets worldwide (Jia 2019). To widely disseminate the results of these genome-wide analyses, the ENIGMA-Vis portal (Shatokhina 2018) provides access to the summary statistics from ENIGMA's GWAS. Hundreds of users have downloaded these data, including researchers in psychiatric and behavioral genetics who study interactions between genes, brain, and behavior. Many researchers have used the summary data to test methods for computing genetic correlations, genomic structural equation modeling (SEM), to relate GWAS findings to maps of gene expression, or to optimize predictive models based on polygenic risk scores. As an example of the impact of ENIGMA on work in related fields, others have used the data to study the overlap between genes affected in *in vitro* models of cell aging with those regulating hippocampal volume.

II. **Structural MRI.** The ENIGMA structural image processing protocols include a wealth of methods for analyzing T1-weighted brain MRI including cortical and subcortical volumes and surface areas, sulcal analysis, longitudinal analysis and vertex-wise subcortical shape analysis.

FreeSurfer, a brain imaging software package developed for analyzing brain MRI scans, is primarily used to preprocess and label neuroanatomical structures in the data (Fischl 2002). Detailed instructions for analysis and quality control for each method may be found on the ENIGMA website (http://enigma.ini.usc.edu/protocols/imaging-protocols/). ENIGMA's Ataxia group has also developed and tested Docker-based pipelines for analyzing cerebellar structure, by meta-analyzing statistical maps across multiple sites and cohorts (Harding 2019a, 2019b).

III. **Diffusion MRI.** ENIGMA's DTI protocols (http://enigma.ini.usc.edu/protocols/dti-protocols/), now used in the largest diffusion MRI studies of 8 different brain disorders (Kelly 2018, van Velzen 2019, Favre 2019, Villalon 2019; Dennis 2018, 2019; Piras 2020, Hatton 2019; Jahanshad 2017, 2013; Acheson 2017, Kochunov 2016, 2015, 2014), allow the pre-processing, quality control, and meta-analysis of data collected with diverse diffusion imaging protocols. The ENIGMA-DTI protocol is based on tract-based spatial statistics and includes steps for motion and eddy currents correction, echo-planar imaging distortion correction and tensor fitting. The ENIGMA-DTI protocol may be found on the ENIGMA website (http://enigma.ini.usc.edu/protocols/dti-protocols/) and is detailed in Jahanshad (2013). This protocol has excellent reproducibility for the analysis of white matter microstructure (Acheson 2017). Recent diffusion MRI studies by ENIGMA also show the benefits of using additional data harmonization methods, such as the batch-effect correction tool ComBat (Fortin 2017; Hatton 2019 compare global analyses of epilepsy data with and without using ComBat, showing its advantages; see also Zhu 2018; Nir 2018 on DTI data harmonization).

IV. **Anatomical Shape.** ENIGMA's Subcortical Shape Analysis toolbox (http://enigma.ini.usc.edu/ongoing/enigma-shape-analysis/; Gutman 2015) has been used to meta-analyze local effects of genetic variation (Roshchupkin 2016), psychiatric and neurological disorders, and modulators of disease, on 3D surface models of subcortical structures. The toolbox has been used to support multi-site analyses of subcortical shape in MDD (Ho 2019), OCD (Fouche 2019), schizophrenia (Gutman 2019), addiction (Chye 2019), 22q11.2 deletion syndrome (Lin 2017; Ching 2019), and Parkinson's disease (Laansma 2020). It has also been used to create surface based maps of SNP effects for genetic loci discovered in ENIGMA's GWAS (Hibar 2015). In recent innovations, deep learning has been adapted to vertex-based data created by the ENIGMA Shape Analysis toolbox to enhance diagnostic classification. For quality control, we have adapted standard deep learning approaches to handle subcortical and cortical meshes (Petrov 2017; Zeng 2020).

V. **EEG.** ENIGMA's EEG working group is currently developing methods for analyzing resting state EEG (Smit 2018). Historically, EEG has used highly calibrated recording systems and strict electrode localization protocols (Jasper 1958), extended to denser systems (Oostenveld and Praamstra 2001). The analysis protocols for the first ENIGMA-EEG article aimed to converge EEG preprocessing and analysis steps as much as possible across the wide variety in electrodes available across the older cohorts. In the future, novel analyses will use higher electrode density recordings to run GWAS for functional connectivity (Smit 2010; Stam 2014), oscillatory dynamics (Linkenkaer-Hansen 2007), and theta-beta ratio (Arns 2013; Smit 2005) and take advantage of higher density recordings by increased cleaning quality and increased local activity detection. To harmonize these analyses, the group is following strict analysis protocols using automated data cleaning steps tested against the still gold standard of visual cleaning in a subset of the data. Subsequent EEG parameter extraction algorithms are then easily applied. Finally, QC

is performed by comparing each value against a value imputed using the high-density electrode scheme. Further in the future, ENIGMA-EEG will perform source localization to increase the specificity of connectivity and reduce spurious effects in scalp recorded signals.

VI. **Resting-State Functional MRI.** Resting-state functional MRI (rs-fMRI) is an approach to understand patterns of synchronized brain activity at rest, which can be further decomposed into networks with known functions (e.g., default mode, salience, attention networks). Measures derived from these networks can be associated with multivariate patterns in other types of images, or with clinical symptoms using methods such as latent factor analyses or canonical covariates analysis (CCA; Adhikari 2019). Harmonized processing of rs-fMRI in ENIGMA has used one of two pipelines thus far: (1) a single modality AFNI-based pipeline that does not require the use of anatomical MRI datasets (Adhikari 2018a,b, 2019); and (2) a pipeline known as fMRIprep+ (Veer 2019), based on the fMRIprep approach (Esteban 2019) that can be used for analysis of multi-site task-based fMRI.

VII. **Multi-site statistics: Meta- and Mega-Analyses, and Machine Learning.** Early work by ENIGMA focused on developing technical approaches to meta-analyze effects of disease or genetic variation on the brain, after performing computations at many remote sites. The results of published meta-analyses were imported into a publicly available online 3D viewer - the ENIGMA Viewer - to help users interactively visualize the effects of disease on various brain measures, overlaid on a 3D brain model (http://enigma-viewer.org/About_the_projects.html; Zhang 2017). ENIGMA's early meta-analyses were extended to 'mega-analyses' (see Boedhoe (2019) for a comparison of the two approaches), in which individual-level data are pooled across sites for more sophisticated multivariate analyses; over 300 such analyses are now underway across ENIGMA working groups. ENIGMA also developed protocols to meta-analyze voxel-based data, including multi-site tensor-based morphometry (TBM; Jahanshad 2019). This technique allows each site to compute statistical models on their own brain template, and then to pool or compare findings after nonlinear registration of group data to a common neuroanatomical template. More recently, projects have begun to perform machine learning on both raw image data and derived data, to build predictive models that can be trained and thoroughly tested on diverse datasets worldwide (Nunes 2018; Bruin 2019).

VIII. **Informatics for Large-Scale Multi-Site Projects.** The organization, management, and tracking of projects and meta data on such a vast scale has benefited from informatics approaches that represent large-scale collaborative studies. One tool being developed is the ENIGMA Organic Data Science framework (Jahanshad 2015), a semantic media wiki based site that integrates information and relationships among co-authors, cohorts, projects, working groups, and the data types and properties relevant to each category. ENIGMA-ODS (Jahanshad 2015) is currently designed only to store meta-data for cohorts, to include, for example, the imaging and genetic data types collected, the number and type of participants, and the scanning locations, allowing for automatic generation of cohort description tables and supplementary information (Jang 2017). Situations encountered throughout ENIGMA analyses fuel continuously updated features in the wiki, necessary before widespread deployment. For example, the continued data collection in some cohorts, as opposed to others, results in a previous version of one cohort's data being used for older projects compared to newer ones; therefore, a "project cohort" page allows for unique subsets of cohorts that were contributed to certain projects to be described and fixed, while allowing the meta-data for the cohort at large to be updated. ENIGMA-ODS provides an

environment for ENIGMA researchers initiating a project to search meta-data for other cohorts with relevant data collection types and recruit researchers to participate in new endeavours. The project proposals also provide an option for registering hypotheses and analysis plans to the research network to ensure proper scientific research guidelines are met. Neuro-DISK (Garijo 2019), an extension of the Automated DIscovery of Scientific Knowledge (DISK) framework (Gil 2016) to support multi-site studies of neuroimaging genetics, is currently underway. This framework provides and implements a detailed description of statistical analyses so that they can be re-run, supplemented, and updated as new data become available. This level of continuous data monitoring and updating supports the growing call for reproducible data science (Gundersen 2018).

IX. **Distributed Computation.** An upcoming innovation in ENIGMA's data analyses includes the use of COINSTAC (Plis 2016, Ming 2017) - a framework that allows computation on distributed brain imaging data. Distributed computation allows a user to compute on remotely stored data, using workflows that can iterate over multiple datasets and servers. This kind of approach helps to coordinate analyses without requiring the data to be centralized in one place; initial tests of COINSTAC in ENIGMA are underway in the schizophrenia, bipolar disorder, and MDD working groups.

The technical scope of ENIGMA is constantly evolving. The last year has seen the creation of working groups to harmonize data from MRS (Bartnik-Olson 2019), and to compare approaches to compute BrainAGE (Han 2019; Lam 2020).

B. Acknowledgements

NJ is supported by R01 AG059874 and R01 MH117601. CRKC is supported by ENIGMA's NIH Big Data to Knowledge (BD2K) initiative U54 EB020403 and T32 Postdoctoral Scholar Fellowship Trainee Grant 5251831121. BMA is supported by NIH grant T32 MH067533. IA is supported by Research Council of Norway (213700, 250358), South-Eastern Norway Regional Health Authority (2012100), Swedish Research Council (K2015-62X-15077-12-3, 2017-90049, FORMAS) and the Kristian Gerhard Jebsen Stiftelsen (SKGJ- MED- 008). AA is supported by University Medical Center Groningen. RRA receives grant research support from NIMH, NIDA, and the Klingenstein Third Generation Foundation. AA holds a Medical Research Council eMedLab Medical Bioinformatics Career Development Fellowship; this work was supported by the Medical Research Council (grant No MR/L016311/1). OAA is supported by Research Council of Norway (223273, 248778, 248980, 249711), South-East Norway Health Authority (2019108) and the Kristian Gerhard Jebsen Stiftelsen (SKGJ- MED- 008). DAB is supported by APA, SAMHSA-State of Calif. JMB-H is supported by the Leiden University Research Profile 'Health, Prevention and the Human Life Cycle' and the Institute of Psychology of Leiden University. CEB is supported by NIH/NIMH grant R01 MH085953, NIH/NIMH grant R01 MH100900, ENIGMA's NIH Big Data to Knowledge (BD2K) initiative U54 EB020403, and SFARI Explorer Award. LAB is supported by NIMH grant F32 MH108311. RMB is supported by 1R56 AG058854. JKB is supported by the EU-AIMS and AIMS-2-TRIALS grants from the Innovative Medicines Initiative Joint Undertaking under grant agreements No 115300, and No 777394, the resources of which are composed of financial contributions from the European Union's Seventh Framework Programme (grant FP7/2007-2013), from the European Federation of Pharmaceutical Industries and Associations companies' in-kind contributions, and from

Autism Speaks. He is further supported by the European Community's Seventh Framework Programme under the grant agreements No 602805 (Project EU-AGGRESSOTYPE), No 602450 (Project EU-IMAGEMEND), No 603016 (project MATRICS), No. 667302 (project CoCA), No 278948 (project TACTICS), No 728018 (project Eat2beNICE), No 643051 (MiND) and No 642996 (BRAINVIEW), and by grants from the Dutch grant system: NWO Large Investment Grant No 1750102007010, ZonMW grant No 60-60600-97-193, and NWO grants No 056-13-015 and No 433-09-242. KC is supported by a National Health and Medical Research Council Career Development Fellowship and an ACURF Program grant by the Australian Catholic University (ACU). CAMC is supported by the European Union's Horizon 2020 research and innovation programme under the Marie Skłodowska-Curie grant agreement No 707404. CAMC and EW are supported by the European Union's Horizon 2020 research and innovation programme (grant agreement No 848158; EarlyCause). RAC is supported by NIAAA, NIA, NIDDK, NIDA. JHC is supported by a UKRI Innovation Fellowship. The Enigma Addictions WG is supported by NIDA grant 1R21 DA038381 and ENIGMA's NIH Big Data to Knowledge (BD2K) initiative U54 EB020403. ELD is supported by K99 NS096116. SD is supported by grants from ENIGMA's NIH Big Data to Knowledge (BD2K) initiative U54 EB020403; the Medical Research Foundation and Medical research council (grant No MR/R00465X/1), the European Union-supported FP6 Integrated Project IMAGEN (Reinforcement-related behavior in normal brain function and psychopathology) (LSHM-CT- 2007-037286), the Bundesministerium für Bildung und Forschung (BMBF grants 01GS08152; 01EV0711; eMED SysAlc01ZX1311A, the Medical Research Council Grant 'c-VEDA' (Consortium on Vulnerability to Externalizing Disorders and Addictions) (MR/N000390/1) and the National Institute for Health Research (NIHR) Biomedical Research Centre at South London and Maudsley NHS Foundation Trust and King's College London. SE is supported by the Deutsche Forschungsgemeinschaft (EH 367/5-1 and SFB 940), the Swiss Anorexia Nervosa Foundation and the Roland Ernst Stiftung. SDB and GF are supported by the European Community's Seventh Framework Programme under the grant agreements No 602407 (Project FemNAT-CD). SEF and CF are supported by the Max Planck Society (Germany). SF is supported by R01 MH113619, and ENIGMA World Aging Center (NIA R56 AG058854), and by the ENIGMA Sex Differences Initiative (R01 MH116147). BF is supported by the Netherlands Organization for Scientific Research (NWO), i.e. the Vici Innovation Program (grant 016-130-669 to BF); additional support was received from the Dutch National Science Agenda for the NWANeurolabNL project (Grant 400 17 602), from the ECNP Network ADHD across the Lifespan, and from the European Community's Horizon 2020 Programme (H2020/2014 – 2020) under grant agreements No 667302 (CoCA) and No 728018 (Eat2beNICE). HPG is supported by R01 DA047119, T32 DA043593, 2P20 GM103644. DCG is supported by R01 MH106324. TPG is supported by The Research Council of Norway (grant No 223273) and South-Eastern Norway Regional Health Authority (grants No 2017112). BAG is supported by ENIGMA's NIH Big Data to Knowledge (BD2K) initiative U54 EB020403, the Alzheimer's Association, and Michael J. Fox Foundation Biomarkers Across Neurodegenerative Diseases (2015). IHH is supported by NHMRC (Fellowship 1106533). FGH is supported by NIH/NINDS/NIA/PA-DOH. MH is supported by Netherlands Organization for Scientific Research (NWO, grant No 91619115). ENIGMA-Plasticity is supported in part by NIH R56 AG058854 (PI PMT; subaward to HEH-P). MJ is supported by K01 MH112774. MK is supported by the Dutch National Science Agenda for the NWANeurolabNL project (grant No 400 17 602). RCK is supported by the NIMH (1R33MH104330). PK is supported by R01 EB015611. IKK is supported by the European Union (ERC Starting grant and ERA-NET Neuron), the German Ministry of Education and Research, and the National Institutes of Health (R01NS100952, U01NS093334. S-LL is supported by K01HD091283. APL is supported by W81XWH-15-1-0412, 5U01NS093334-02, 1R01NS100952-01A1,

AARG-17-533222 and Women's Brain Initiative. MWL is supported by VA BLR&D I01BX003477 PI Logue, 1R01 MH111671 PI Morey. FM is partially supported by NIH grant NIMH R21 MH115327-01. SM is supported by NIH/NIDA 1R01DA047119-01. CRM is supported by NIH/NINDS R01 NS065838 and R21 NS107739. ABM is supported by ENIGMA grants. SEM is supported by Australian National Health and Medical Research Council APP1103623 and APP1158127. GM is supported by a Sir Henry Dale Fellowship, jointly supported by the Wellcome Trust and the Royal Society (#202397/Z/16/Z). RAM is supported by US Department of Veterans Affairs (VA) Mid-Atlantic Mental Illness Research, Education, and Clinical Center (MIRECC) core funds, US Department of Veterans Affairs (VA) Office of Research and Development (5I01CX000748-01, 5I01CX000120-02), National Institute for Neurological Disorders and Stroke (R01 NS086885), National Institute for Mental Health (R01MH111671). SCM would like to thank the BOF (University of Ghent) for supporting his work on transgender and PTSD research (01J05415 and 01IO0318). PM is supported by NIH R01 NS060776, NIH RC2 NS069409, TBI Endpoints Development (TED): DoD W81XWH-14-2-0176, TRACK-TBI: NIH U01 NS086090. TMN is supported by T32 AG058507. DP is supported by NIMH Intramural Research Program Project ZIAMH002781. FP is supported by RF1 5351832013. JDR is supported by UK Medical Research Council, The Bluefield Project, NIHR, and the Association for Frontotemporal Degeneration. PGS is supported by the German Research Foundation (DFG, SA 1358/2-1) and the Max Planck Institute of Psychiatry, Munich. LS is supported by a NHMRC Career Development Fellowship (1140764). GSc is supported by Horizon 2020 supported ERC Advanced Grant ‘STRATIFY’ (Brain network based stratification of reinforcement-related disorders) (695313) and Medical Research Council Grant 'c-VEDA’ (Consortium on Vulnerability to Externalizing Disorders and Addictions) (MR/N000390/1). MSS is supported by SC CTSI (NIH/NCRR/NCATS) 5KL2TR000131-05, NIH Loan Repayment Program1 L30 CA209248-01, American Cancer Soc. Institutional Research Grant Wright Foundation Pilot Award; Radiological Society of North America (RSNA) Resident/Fellow Grant, USC Center for Imaging Acquisition grant. SMS is supported by Epilepsy Society; this work was partly undertaken at UCLH/UCL, which received a proportion of funding from the Department of Health’s NIHR Biomedical Research Centres funding scheme. IES is supported by the Research Council of Norway (223273), the Kristian Gerhard Jebsen Stiftelsen (SKGJ- MED-008) and South-Eastern Norway Regional Health Authority (grant No 2020060). DJS is supported by the South African Medical Research Council. JLS is supported by the NIH grants R01 MH118349, R00MH102357, and ENIGMA's NIH Big Data to Knowledge (BD2K) initiative U54 EB020403. DFT is supported by Chronic Effects of Neurotrauma Consortium. YDvdW is supported by NIH R56 ENIGMA World Aging. TGMvE is supported by ENIGMA's NIH Big Data to Knowledge (BD2K) initiative U54 EB020403 and R01MH116147. IMV is supported by German Research Foundation (Deutsche Forschungsgemeinschaft) grants ER724/4-1 and WA1539/11-1. HW is supported by a NARSAD Distinguished Investigator Grant Schizophrenia (grant No 26985) and a specific ENIGMA grant of the German Research Foundation (WA 1539/11-1). JEV-R and NS are supported by ENIGMA's NIH Big Data to Knowledge (BD2K) initiative U54 EB020403. CDW is supported by Health Research Board of Ireland PhD. EAW is supported by the Department of Defense, Chronic Effects of Neurotrauma Consortium (CENC) Award W81XWH-13-2-0095, VA 1I01RX001062. The PRISM project (www.prism-project.eu) leading to this manuscript has received funding from the Innovative Medicines Initiative 2 Joint Undertaking under grant agreement No 115916. This Joint Undertaking receives support from the European Unions Horizon 2020 research and innovation program and EFPIA. WBB is supported by Netherlands Organization for Scientific Research (NWO/ZonMW Vidi 016.156.318). JC is supported by NSF-1302755, NSF DBI-1260795, and NSF CNS-1531491. YC is supported by the Monash Bridging Postdoctoral Fellowship. UD is supported by the

German Research Foundation (DFG, grant No FOR2107 DA1151/5-1 and DA1151/5-2 to UD; SFB-TRR58, Projects C09 and Z02 to UD) and the Interdisciplinary Center for Clinical Research (IZKF) of the medical faculty of Münster (grant No Dan3/012/17 to UD). THan was supported by grants the German Research Foundation (DFG grants HA7070/2-2, HA7070/3, HA7070/4 to TH) and from IMF (Innovative Medizinische Forschung) of the medical faculty of Münster (grant OP121710 to TH and Nils Opel). GD is supported by the European Research Council and Science Foundation Ireland European Research Council (grant No 677467), and the Science Foundation Ireland (16/ERCS/3787). LTE is supported by Desert Pacific Mental Illness Research Education and Clinical Center. SVF is supported by the European Union's Horizon 2020 research and innovation programme grant agreement No 667302. PF and JH are supported by Agence Nationale pour la Recherche (ANR-11-IDEX-0004 Labex BioPsy, ANR-10-COHO-10-01 psyCOH), Fondation pour la Recherche Médicale (Bioinformatique pour la biologie 2014) and the Fondation de l'Avenir (Recherche Médicale Appliquée 2014). CAF is supported by NIH Intramural Research Program. TF is supported by the Science Foundation Ireland (SFI). KLG is supported by APP1173025. YG is supported by NIH grant 1R01AG059874-01, DARPA grant FA8750-17-C-0106, NIH grant 1R01 GM117097-01, DARPA grant W911NF-15-1-0555, and a grant from The Kavli Foundation. THaj is supported by grants from the Canadian Institutes of Health Research (grant No 106469, 142255), the Nova Scotia Health Research Foundation, Brain & Behavior Research Foundation (formerly NARSAD) 2007 Young Investigator and 2015 Independent Investigator Awards to TH, and the Ministry of Health, Czech Republic (grants Number 16-32791A, 16-32696A). TCH is supported by the NIMH (K01 MH117442). LH is supported by European Research Council (grant No 677467), and the Science Foundation Ireland (16/ERCS/3787). II is supported by 1R03 DA25796-1 –NIDA, K23 PA-00-003 NIDA AACAP. TJ is supported by 111 Project (B18015), the key project of Shanghai Science & Technology (16JC1420402), Shanghai Municipal Science and Technology Major Project (2018SHZDZX01), NSFC (91630314, 81801773), Shanghai Pujiang Project (18PJ1400900) and ZJLab. JL is supported by the European Research Council ERC-ADG-2014-671084-INSOMNIA. UL is supported by Deutsche Forschungsgemeinschaft (DFG, German Research Foundation) – project No 44541416 – TRR 58 (projects C09 and Z02 to UL). AN is supported by Killam Trust, Nova Scotia Health Research Foundation, and Genome Atlantic. JON is supported by NIMH R01MH081864, NIMH R01MH085900. GSp, FP and FP from the Santa Lucia Foundation in Rome, Italy are funded by the Italian Ministry of Health grants RC12-13-14-15-16-17-18-19/A. CS-M is supported by Miguel Servet's contract (ISCIII grant CPII16//00048) from the Carlos III Health Institute. LT is supported by NIH U01 MH109985. EJWVS is supported by European Research Council ERC-ADG-2014-671084-INSOMNIA. GAvW is supported by ZonMW Vidi 016.156.318. LW is supported by 1 U01 MH097435, 1 R01 EB020062, NSF SP0037646, NSF BCS 1734853. MJW is supported ENIGMA's NIH Big Data to Knowledge (BD2K) initiative U54 EB020403 sub-award 56929223, NICHD R01 HD050735, NHMRC (Australia) 486682, 1009064. J-YY is supported by the Basic Science Research Program through the National Research Foundation of Korea (NRF) supported by the Ministry of Education (NRF-2017R1D1A1B03028464). GZ is supported by NSF-1302755 and NSF DBI-1260795. DSP is supported by NIMH-Intramural Research Program Project No ZIA MH-002781. ARM is supported by R01MH101512, R01HD086704 and W81XWH-17-2-0052. SHIP is part of the Community Medicine Research net of the University of Greifswald, Germany, which is supported by the Federal Ministry of Education and Research (grant No 01ZZ9603, 01ZZ0103, and 01ZZ0403), the Ministry of Cultural Affairs and the Social Ministry of the Federal State of Mecklenburg-West Pomerania. Genome-wide SNP typing in SHIP and MRI scans in SHIP and SHIP-TREND have been

supported by a joint grant from Siemens Healthineers, Erlangen, Germany and the Federal State of Mecklenburg-West Pomerania.

C. Conflicts of Interest and Disclosures

PMT and NJ are MPIs of a research related grant from Biogen, Inc., for research unrelated to the contents of this manuscript. CRKC and TMN are partially funded by a Biogen Grant (to NJ and PMT) for research unrelated to the contents of this manuscript. RRA is a partner of WISER Systems, LLC. OAA is a consultant to HealthLytix, Speakers honorarium from Lundbeck. JKB has been in the past years a consultant to / member of advisory board of / and/or speaker for Shire, Roche, Medice, and Servier. He is not an employee of any of these companies, and not a stock shareholder of any of these companies. He has no other financial or material support, including expert testimony, patents, royalties. GF is an Editor of European Child & Adolescent Psychiatry. BF has received educational speaking fees from Medice. DPH is a full-time employee of Genentech, Inc. IKK's spouse is an employee of Siemens Healthineers, and IKK receives funding from Abbott and Expesicor. APL is a consultant for Agios, Biomarin, Moncton MRI, and co-founder of BrainSpec, Inc. CDW is an employee of Biogen, Inc. In the past year, SVF has received income, potential income, travel expenses continuing education support and/or research support from Tris, Otsuka, Arbor, Ironshore, Shire, Akili, Enzymotec, Sunovion, Supernus and Genomind. With his institution, he has US patent US20130217707 A1 for the use of sodium-hydrogen exchange inhibitors in the treatment of ADHD. In previous years, he received support from: Shire, Ironshore, Neurovance, Alcobra, Rhodes, CogCubed, KemPharm, Enzymotec, Akili, Neurolifesciences, Lundbeck/Takeda, Otsuka, McNeil, Janssen, Novartis, Pfizer and Eli Lilly. He also receives royalties from books published by Guilford Press: Straight Talk about Your Child's Mental Health, Oxford University Press: Schizophrenia: The Facts and Elsevier: ADHD: Non-Pharmacologic Interventions. He is principal investigator of www.adhdinadults.com. HJG has received travel grants and speakers honoraria from Fresenius Medical Care, Neuraxpharm and Janssen Cilag as well as research funding from Fresenius Medical Care. II received a DSMC Lundbeck honorarium and receives support from the NIDA Stanley Foundation for research. In the past 3 years, DJS has received research grants and/or consultancy honoraria from Lundbeck and Sun. He is supported by the SA Medical Research Council. RCK is a Co-I on a research grant from Nestle/Wyeth for research unrelated to the contents of this manuscript. The remaining authors have no relevant financial disclosures to report. RB's work is part of the Community Medicine Research net (CMR) of the University of Greifswald, Germany. The CMR encompasses several research projects that share data from the population-based SHIP project (http://ship.community-medicine.de). NH is supported by the Joint Project Siemens AG, Erlangen and the federal state of Mecklenburg-Vorpommern, Germany. HV is supported by SHIP is part of the Research Network Community Medicine of the University Medicine Greifswald, which is supported by the German Federal State of Mecklenburg-Vorpommern.

References:

Acheson, A. et al. Reproducibility of tract-based white matter microstructural measures using the ENIGMA-DTI protocol. *Brain Behav.* 7, e00615 (2017).

Adhikari, B. M. et al. Comparison of heritability estimates on resting state fMRI connectivity phenotypes using the ENIGMA analysis pipeline. *Hum. Brain Mapp.* 39, 4893–4902 (2018).

Adhikari, B. M. et al. Effects of ketamine and midazolam on resting state connectivity and comparison with ENIGMA connectivity deficit patterns in schizophrenia. *Hum. Brain Mapp.* 1–12 (2019).

Adhikari, B. M. et al. Heritability estimates on resting state fMRI data using ENIGMA analysis pipeline. *Pac. Symp. Biocomput.* 23, 307–318 (2018).

Arns, M., Conners, C. K. & Kraemer, H. C. A decade of EEG Theta/Beta Ratio Research in ADHD: a meta-analysis. *J. Atten. Disord.* 17, 374–83 (2013).

Bartnik-Olson, B. et al. The Clinical Utility of Magnetic Resonance Spectroscopy in Traumatic Brain Injury: Recommendations from the ENIGMA MRS Working Group. *Brain Imaging Behav*. (Special Issue on ENIGMA Brain Injury). Preprint at https://doi.org/10.31234/osf.io/gesvh (2019) (submitted).

Boedhoe, P. S. W. et al. An Empirical Comparison of Meta- and Mega-Analysis with Data from the ENIGMA Obsessive-Compulsive Disorder Working Group. *Front. Neuroinf.* 12, 102 https://doi.org/10.3389/fninf.2018.00102 (2019).

Bruin, W. B. et al. Structural neuroimaging biomarkers for obsessive-compulsive disorder in the ENIGMA-OCD consortium: medication matters. Preprint at https://doi.org/10.1101/19012567 (2019).

Ching, C. R. K. et al. Mapping Subcortical Brain Alterations in 22q11.2 Deletion Syndrome: Effects of Deletion Size and Convergence with Idiopathic Neuropsychiatric Illness. *American J. of Psychiatry*, appiajp201919060583, doi:10.1176/appi.ajp.2019.19060583 (2020).

Chye, Y. et al. Subcortical surface morphometry in substance dependence: an ENIGMA addiction working group study. *Addiction Biol*. e12830 (2019).

Chye, Y. et al. Subcortical surface morphometry in substance dependence: an ENIGMA addiction working group study. *Addiction Biol*. e12830 (2019).

Dennis, E. L. et al. Altered white matter microstructural organization in posttraumatic stress disorder across 3,047 adults: results from the PGC-ENIGMA PTSD Consortium. Mol. J. Psychiatry https://doi.org/10.1038/s41380-019-0631-x (2019).

Dennis, E. L. et al. ENIGMA pediatric msTBI: preliminary results from meta analysis of diffusion MRI. In 14th International Symposium on Medical Information Processing and Analysis. (International Society for Optics and Photonics, 2018).

Esteban, O. et al. fMRIPrep: a robust preprocessing pipeline for functional MRI. Nat. Methods 16, 111–116 (2019).

Favre, P. et al. Widespread white matter microstructural abnormalities in bipolar disorder: evidence from mega- and meta-analyses across 3033 individuals. *Neuropsychopharmacology* 44, 2285–2293 (2019).

Fischl, B., et al. Whole brain segmentation: automated labeling of neuroanatomical structures in the human brain. *Neuron*, *33*(3), 341–355 (2002).

Fortin, J. P. et al. Harmonization of multi-site diffusion tensor imaging data. *NeuroImage* 161, 149–170 (2017).

Fouche, J.-P. et al. Shape analysis of subcortical structures in obsessive compulsive disorder: a multi-site analysis of the OCD Brain Imaging Consortium. (2020) (in preparation).

Garijo, D. et al. Towards Automated Hypothesis Testing in Neuroscience. In Gadepally, V., Mattson, T., Stonebraker, M., Wang, F., Luo, G., Laing, Y., and Dubovitskaya, A., editor(s), Heterogeneous Data Management, Polystores, and Analytics for Healthcare, pages 249-257, Cham. Springer International Publishing (2019).

Gil, Y. et al. Automated hypothesis testing with large scientific data repositories. In Proceedings of the Fourth Annual Conference on Advances in Cognitive Systems (ACS) (2016).

Grasby, K. L. et al. The genetic architecture of the human cerebral cortex. *Science*. (2020) (in press). Preprint at https://doi.org/10.1101/399402.

Gundersen, O. E., Gil, Y. & Aha, D. W. On reproducible AI: towards reproducible research, open science, and digital scholarship in AI Publications. AI Mag. 39, 56–68 (2018).

Gutman, B. A. et al. A Meta-Analysis of Deep Brain Structural Shape Abnormalities in 2,763 Individuals with Schizophrenia Compared to 3,768 Healthy Volunteers via the ENIGMA Consortium. To be submitted to *Biol. Psychiatry.* (2020) (to be submitted).

Gutman, B. A. et al. Medial demons registration localizes the degree of genetic influence over subcortical shape variability: An N=1480 meta analysis. in Biomedical Imaging (ISBI), 2015 IEEE 12th International Symposium 1402–1406(IEEE, 2015).

Han, L. K. M. et al. Brain aging in major depressive disorder: results from the ENIGMA Major Depressive Disorder working group. *bioRxiv*. https://doi.org/10.1101/560623 (2019).

Harding, I. et al. Brain atrophy in Friedreich ataxia preferentially manifests in cerebellar and cerebral motor areas: results from the ENIGMA-Ataxia consortium. Presented at the International Ataxia Research Conference Nov 14–16, abstract 169 (Washington, DC, 2019).

Harding, I. et al. The spatial distribution of cerebellar and brainstem structural abnormalities in SCA1, 2, 3, and 6 from the ENIGMA-Ataxia consortium. Presented at the International Ataxia Research Conference Nov 14–16, abstract 170 (Washington, DC, 2019).

Hatton, S. et al. White matter abnormalities across different epilepsy syndromes in adults: an ENIGMA Epilepsy study. Brain. (under review). Preprint on bioRxiv 2019.12.19.883405; doi: https://doi.org/10.1101/2019.12.19.883405 (2019).

Hibar, D. P. et al. Common genetic variants influence human subcortical brain structures. *Nature* 520, 224–229 (2015).

Hibar, D. P. et al. Common genetic variants influence human subcortical brain structures. *Nature* 520, 224–229 (2015).

Hofer, E. et al. Genetic determinants of cortical structure (thickness, surface area and volumes) among disease free adults in the CHARGE Consortium. *bioRxiv*. https://doi.org/10.1101/409649 (2019).

Jahanshad, N. al. Genome-wide scan of healthy human connectome discovers SPON1 gene variant influencing dementia severity. *Proc. Natl Acad. Sci*. USA 110, 4768–4773 (2013).

Jahanshad, N. et al. Supporting the Consortium for Enhancing NeuroImaging Genetics through Meta-Analysis (ENIGMA) through the Organic Data Science Framework. Presented at the Conference on Science of Team Science (SciTS) (Bethesda, MD, June 2015).

Jahanshad, N. et al. Multi-site meta-analysis of morphometry. IEEE/ACM Trans. Computational Biol. Bioinforma. 16, 1508–1514 (2019).

Jang, M. H. et al. Towards Automatic Generation of Portions of Scientific Papers for Large Multi-Institutional Collaborations Based on Semantic Metadata. Proceedings of the Workshop on Enabling Open Semantic Science, Co-located with the Sixteenth International Semantic Web Conference (ISWC) (Vienna, Austria, October 2017).

Jasper, H. H. The ten-twenty electrode system of the international federation. *Electroencephalogr. Clin. Neurophysiol.* 10, 371–375 (1958).

Jia, T. et al. Epigenome-wide meta-analysis of blood DNA methylation and its association with subcortical volumes: findings from the ENIGMA Epigenetics Working Group. *Mol Psychiatry*. doi: 10.1038/s41380-019-0605-z (2019).

Kelly, S. et al. Widespread white matter microstructural differences in schizophrenia across 4322 individuals: results from the ENIGMA Schizophrenia DTI Working Group. *Mol. Psychiatry* 23, 1261–1269 (2018).

Kochunov, P. et al. Heritability of fractional anisotropy in human white matter: a comparison of Human Connectome Project and ENIGMA-DTI data. *NeuroImage* 111, 300–311 (2015).

Kochunov, P. et al. Heterochronicity of white matter development and aging explains regional patient control differences in schizophrenia. *Hum. Brain Mapp*. 37, 4673–4688 (2016).

Kochunov, P. et al. Multi-site study of additive genetic effects on fractional anisotropy of cerebral white matter: comparing meta and mega analytical approaches for data pooling. *NeuroImage* 95, 136–150 (2014).

Laansma, M. et al. Bidirectional Changes in Subcortical Shape Derived Local Thickness Measures: an ENIGMA-Parkinson's Disease Mega-Analysis (N=1649). Presented at the 15th International Conference of Alzheimer's and Parkinson's Diseases April 2–5, abstract 701 (Vienna, 2020).

Lam, P., Zhu, A., Salminen, L., Jahanshad, N. & Thompson, P. M. Predicting Brain Age from Structural MRI using Deep Learning and Information Theoretic Divergence Measures. Accepted at ISBI 2020 (Iowa City, IA, 2020).

Lin, A. et al. Mapping 22q11.2 gene dosage effects on brain morphometry. *J. Neurosc.* 37, 6183–99 (2017).

Linkenkaer-Hansen, K. et al. Genetic contributions to long-range temporal correlations in ongoing oscillations. *J. Neurosci. 27*, 13882–9 (2007).

Ming, J. et al. COINSTAC: decentralizing the future of brain imaging analysis. F1000Res 2017 6, 1512 (2017).

Nir, T. M. et al. Effects of diffusion MRI model and harmonization on the consistency of findings in an international multi-cohort HIV neuroimaging study. In: Bonet-Carne E., Grussu F., Ning L., Sepehrband F., Tax C. (eds) Computational Diffusion MRI. MICCAI 2019. Mathematics and Visualization. Springer, Cham (2019).

Oostenveld, R. & Praamstra, P. The five percent electrode system for highresolution EEG and ERP measurements. *Clin. Neurophysiol.* 112, 713–9 (2001).

Petrov, D. et al. Machine learning for large-scale quality control of 3D shape models in neuroimaging. *bioRxiv*. https://doi.org/10.1101/166496 (2017).

Piras, F. et al. Selective white matter microstructure and its relation to clinical features of obsessive-compulsive disorder: findings from the ENIGMA OCD Working Group. *bioRxiv*. https://doi.org/10.1101/855916 (2019).

Plis, S. M. et al. COINSTAC: a privacy enabled model and prototype for leveraging and processing decentralized brain imaging data. *Front. Neurosci.* 10, 365 (2016)

Roshchupkin, G. V. et al. Heritability of the shape of subcortical brain structures in the general population. *Nat. Commun*. 7, 13738 (2016).

Satizabal, C. L. et al. Genetic architecture of subcortical brain structures in 38,854 individuals worldwide. *Nat Genet*, **51**(11), 1624-1636 (2019).

Shatokhina, N. et al. ENIGMA-Vis: A Portal to View Genetic Effects on the Human Brain Based on Large-Scale GWAS. Presented at the 24th Organization of Human Brain Mapping Annual Meeting Jun 17–21, abstract (Singapore, 2018).

Shatokhina, N. et al. ENIGMA-Vis: A Portal to View Genetic Effects on the Human Brain Based on Large-Scale GWAS. Presented at the 24th Organization of Human Brain Mapping Annual Meeting Jun 17–21, abstract 1546 (Singapore, 2018).

Villalón-Reina, J. E. et al. Altered White Matter Microstructure in 22q11.2 Deletion Syndrome: A Multi-Site Diffusion Tensor Imaging Study. Mol. Psychiatry https://doi.org/10.1038/s41380-019-0450-0 (2019).

Zeng, L.-L. et al. Machine learning on vertex-wise brain shape metrics improves the diagnostic classification of bipolar disorders. To be presented at the 75th SOBP 2020 Annual Meeting, Apr 30-May 2, abstract (New York City, 2020).

Zhang, G. et al. ENIGMA-Viewer: interactive visualization strategies for conveying effect sizes in meta-analysis. *BMC Bioinforma.* 18, 253 (2017).

Zhu, A. H., Moyer, D. C., Nir, T. M., Thompson, P. M. & Jahanshad, N. Challenges and opportunities in diffusion MRI data harmonization. In: Bonet-Carne E., Grussu F., Ning L., Sepehrband F., Tax C. (eds) Computational Diffusion MRI. MICCAI 2019. Mathematics and Visualization. Springer, Cham (2019).
